# RNA dependent suppression of C9orf72 ALS/FTD associated neurodegeneration by Matrin-3

**DOI:** 10.1186/s40478-020-01060-y

**Published:** 2020-10-31

**Authors:** Nandini Ramesh, Elizabeth L. Daley, Amanda M. Gleixner, Jacob R. Mann, Sukhleen Kour, Darilang Mawrie, Eric N. Anderson, Julia Kofler, Christopher J. Donnelly, Evangelos Kiskinis, Udai Bhan Pandey

**Affiliations:** 1 Department of Pediatrics, Children’s Hospital of Pittsburgh, University of Pittsburgh Medical Center, Pittsburgh, PA USA; 2 Department of Human Genetics, School of Public Health, School of Public Health, University of Pittsburgh, Pittsburgh, PA USA; 3 The Ken & Ruth Davee Department of Neurology, Northwestern University Feinberg School of Medicine, Chicago, IL USA; 4 Department of Neurobiology, University of Pittsburgh School of Medicine, Pittsburgh, PA USA; 5 LiveLikeLou Center for ALS Research, University of Pittsburgh Brain Institute, Pittsburgh, PA USA; 6 Department of Pathology, University of Pittsburgh, Pittsburgh, PA USA; 7 Department of Physiology, Northwestern University Feinberg School of Medicine, Chicago, IL USA; 8 Simpson Querrey Institute, Northwestern University, Chicago, IL 60611 USA

## Abstract

The most common genetic cause of amyotrophic lateral sclerosis (ALS) is a GGGGCC (G4C2) hexanucleotide repeat expansions in first intron of the *C9orf72* gene. The accumulation of repetitive RNA sequences can mediate toxicity potentially through the formation of intranuclear RNA foci that sequester key RNA-binding proteins (RBPs), and non-ATG mediated translation into toxic dipeptide protein repeats. However, the contribution of RBP sequestration to the mechanisms underlying RNA-mediated toxicity remain unknown. Here we show that the ALS-associated RNA-binding protein, Matrin-3 (MATR3), colocalizes with G4C2 RNA foci in patient tissues as well as iPSC-derived motor neurons harboring the *C9orf72* mutation. Hyperexpansion of C9 repeats perturbed subcellular distribution and levels of endogenous MATR3 in C9-ALS patient-derived motor neurons. Interestingly, we observed that ectopic expression of human MATR3 strongly mitigates G4C2-mediated neurodegeneration in vivo. MATR3-mediated suppression of C9 toxicity was dependent on the RNA-binding domain of MATR3. Importantly, we found that expression of MATR3 reduced the levels of RAN-translation products in mammalian cells in an RNA-dependent manner. Finally, we have shown that knocking down endogenous *MATR3* in C9-ALS patient-derived iPSC neurons decreased the presence of G4C2 RNA foci in the nucleus. Overall, these studies suggest that MATR3 genetically modifies the neuropathological and the pathobiology of *C9orf72* ALS through modulating the RNA foci and RAN translation.

## Introduction

Amyotrophic lateral sclerosis (ALS) is a neurodegenerative disorder that is characterized by degeneration of upper and lower motor neurons, leading to progressive atrophy and weakness in skeletal muscles, eventually resulting in death due to respiratory failure [1, 2]. An expansion of hexanucleotide GGGGCC (G4C2) repeat within the first intron of C9orf72 gene is the most common cause of familial ALS as well as frontotemporal dementia (FTD), commonly termed as C9-ALS/FTD [3, 4]. While unaffected individuals carry less than 10 G4C2 repeats (3,4), C9-ALS/FTD patients carry anywhere between 30 and 1600 repeats [3, 5].

Extensive investigation into the mechanisms underlying C9-ALS has identified three potentially pathogenic mechanisms resulting from the G4C2 hexanucleotide repeat expansion (G4C2-HRE) [6–8]—(1) Loss-of-function of endogenous C9orf72 protein that could, in turn, affect endosomal trafficking and autophagy pathways [9, 10]; (2) Gain-of-function RNA-toxicity that arises from transcription of G4C2 repeats in sense and antisense direction [11, 12]; and, (3) Gain-of-function protein toxicity caused by repeat associated non-ATG (RAN) translation of G4C2-HRE RNA to produce toxic dipeptide repeat products (DPRs) [12–15]. Both gain-of-function models are supported by observations of pathologic aggregation of G4C2-HRE RNA into intranuclear RNA foci and cytoplasmic inclusions of the DPRs in patient neurons [13, 14, 16, 17]. While these mechanisms have been proposed alternately and have been independently investigated, they are not necessarily mutually exclusive and could be acting synergistically [18].

Accumulation of repeat-bearing RNA in the neurons and glia is also observed in other neurological disorders caused by intronic repeat expansions, such as myotonic dystrophy and fragile X tremor ataxia syndrome, are also characterized by RNA accumulation or RNA foci [19, 20]. The accumulation of expanded repeat-containing RNA transcripts, including G4C2 RNA foci, sequesters essential RNA-binding proteins (RBPs) [11, 21–23], thus leading to a potential loss-of-function of these proteins and, consequently, dysregulation of RNA metabolism [11, 12]. Thus, identification of these RBPs might be critical for understanding the mechanism of C9orf72-mediated neurodegeneration, and possibly serve as therapeutic targets. Matrin-3 (MATR3), an ALS-associated RBP, has been shown to interact with the G4C2 repeat RNA in vitro [24, 25] suggesting a potential functional relationship between them. MATR3 is an essential nuclear matrix protein that, in addition to maintaining the fibrogranular nuclear matrix network, is involved in regulating post-transcriptional RNA processing, including alternative splicing, mRNA stability and mRNA export [26–29]. Moreover, pathogenic mutations in *MATR3* have been discovered in a subset of familial and sporadic ALS cases [30–33], and MATR3-positive cytoplasmic inclusions are widely found in post-mortem brain tissue of C9-ALS and sporadic ALS patients [30, 34]. However, the disease-relevance and the molecular mechanisms underlying these observations is yet unclear.

Here, we demonstrate that MATR3 is an important component of C9orf72-associated disease neuropathology in C9-ALS patient postmortem brain tissues and iPSC motor neurons. We show colocalization between MATR3 and pathogenic G4C2 foci in C9-ALS patient-derived neurons and in post-mortem brain tissues. Additionally, we found that MATR3 sub-cellular localization and levels are altered in C9-ALS patient neurons. Furthermore, we show that ectopic expression of MATR3 strongly suppressed G4C2-HRE toxicity in *Drosophila*, mediated by its RNA-binding domain. In addition, genetic manipulation of MATR3 levels modulated the RAN-translation product levels and RNA foci in mammalian cells and C9-ALS patient-derived motor neurons, respectively. Collectively, our data presents evidence that MATR3 is a novel genetic modifier of neuropathologies and motor dysfunctions associated with G4C2 repeats through modulating the RNA foci and RAN translation products.

## Results

### Pathologic G4C2 RNA foci sequester MATR3 protein in C9-ALS patient neurons

**Fig. 1:**
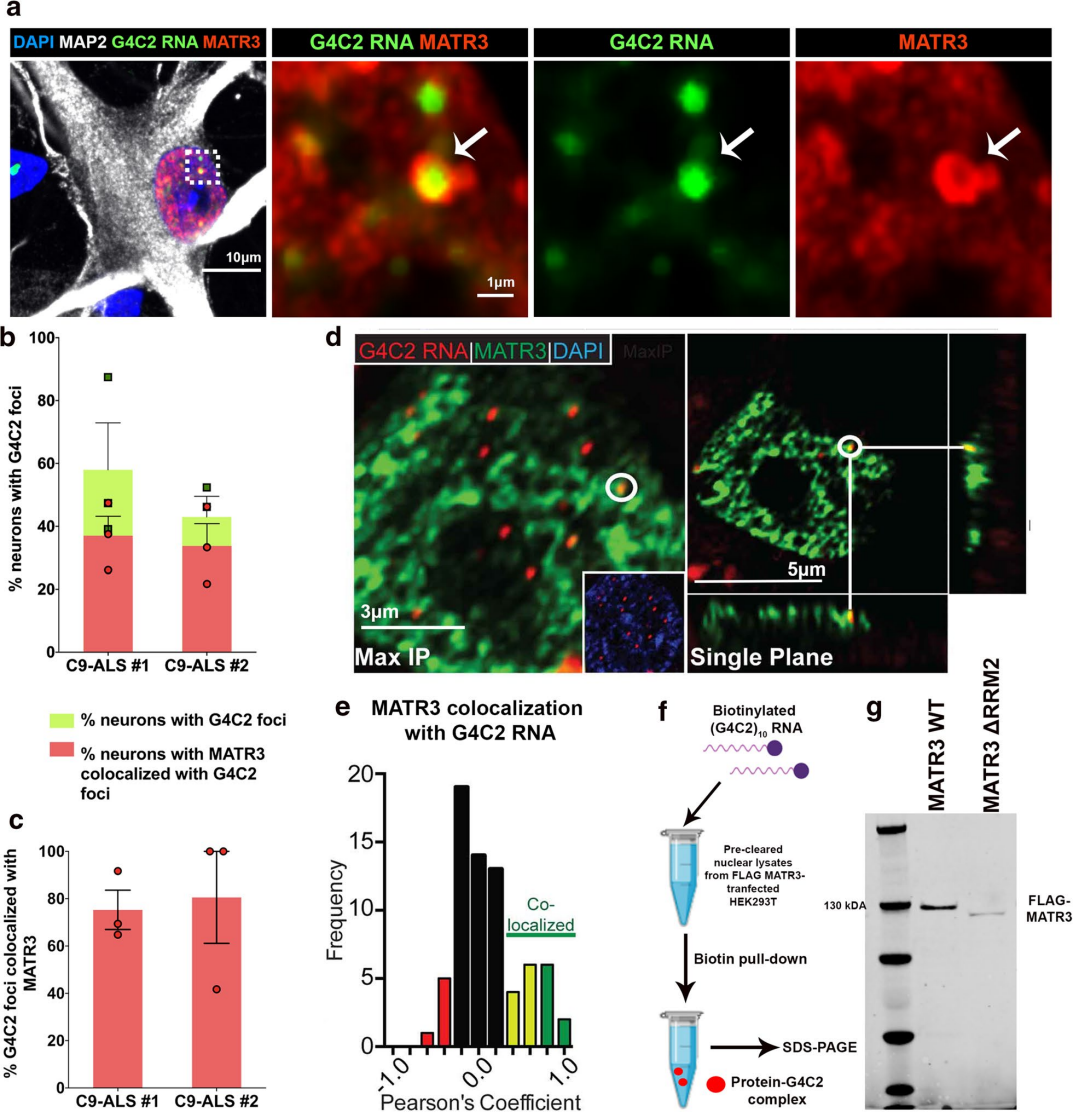
MATR3 colocalizes with pathogenic G4C2 RNA foci in C9-ALS iPSC-derived neurons and in post-mortem brain tissue. **a** Representative confocal image of colocalization (white arrows) between G4C2 RNA foci (green) and MATR3 protein (red) in C9-ALS patient-derived iPSCs differentiated into motor neurons (iPSC-MN), indicated by MAP2 staining (grey). Dotted-white box represents a single G4C2 foci that colocalized with MATR3 and represented in high magnification images to the right. **b** Quantification of percentage neurons that are positive for G4C2 foci (green bars) in two independent C9-ALS patient iPSC-MNs. Average  % neurons that are positive for G4C2 foci in C9-ALS #1 = 58% and in C9-ALS #2 = 48%. Among them, over half of the neurons also showed co-localization between MATR3 and G4C2 RNA foci (red bars). n = 3 differentiations. **c** Quantification of percentage G4C2 foci per neuron that colocalized with MATR3. Average  % G4C2 foci that colocalized with MATR3 in C9-ALS #1 = 75% and in C9-ALS #2 = 80%. n = 3 differentiations. **d** Representative images of immunohistochemical analysis of MATR3 signal (green) in motor cortex neurons from and C9-ALS patient tissue. RNA FISH analysis performed to examine co-localization between G4C2 RNA foci (red) and MATR3 (green) in nuclei of C9-ALS patient tissue cells. Maximum intensity projection (left) and single plane (right) representative images are shown. Inset within image on left represents overlay between Dapi (blue) and G4C2 foci (red). **e** Moderate (yellow) to strong (green) Pearson’s coefficients indicate co-localization between RNA foci and MATR3 signal. n = 70 G4C2 RNA foci from 3 C9-ALS patient tissues. **f** Diagrammatic representation of (G4C2) × 10 pull-down assay. Nuclear lysates from HEK293T cells transiently transfected with either FLAG-MATR3 or FLAG-MATR3-ΔRRM2 were incubated with biotinylated G4C2_10_ RNA. The protein-G4C2 RNA complex was pulled down with streptavidin and then separated by SDS-PAGE. **g** Immunoblot of biotin-G4C2_10_ pull-down fraction probed for FLAG-MATR3 showed physical interaction between MATR3 and G4C2 RNA. The interaction was moderately diminished between MATR3-ΔRRM2 and G4C2 RNA. Error bars indicate S.E.M

Proteomic studies have suggested physical interaction between G4C2 RNA and MATR3 [24, 25], however, the functional significance of this interaction in unclear. This led us to investigate relationship between MATR3 and G4C2 foci localization in neurons. To that end, we utilized C9-ALS patient-derived iPSCs that were further differentiated into motor neurons, hereby referred to as C9 iPSC-MN. Using fluorescent in situ hybridization (FISH) coupled with immunocytochemistry, we analyzed the nuclei of these cells and observed colocalization between G4C2 RNA foci and MATR3 in two independent C9-ALS patient-derived iPSC-MNs (Fig. 1a) (Additional file 1: Figure S1A). We also found that the apparent colocalization between G4C2 foci occurred with both punctate (Fig. 1a) and diffuse (Additional file 2: Figure S2A) forms of MATR3 within the nuclei of C9 iPSC-MNs. Quantitative analysis revealed that about one-third of neurons exhibited colocalization between G4C2 RNA foci and MATR3 (Fig. 1b), within which close to 80% of foci colocalized with MATR3 (Fig. 1c). Conversely, across two C9-ALS iPSC MNs, 14% and 61% of MATR3 puncta, respectively, colocalized with G4C2 RNA, while a significant portion of MATR3 puncta occurred independent of G4C2 foci (Additional file 2: Figure S2B). To further validate G4C2 RNA and MATR3 colocalization in end-stage disease condition, we analyzed motor cortex sections of post-mortem brain tissues and found colocalization between G4C2 RNA and MATR3 in the neurons in C9-ALS patient brains (Fig. 1d, e). The neurons in the post-mortem brain tissue from healthy controls remained devoid of any G4C2-positive foci (Additional file 1: Figure S1B).

To confirm that colocalization is due to physical interaction between MATR3 and G4C2 RNA, we performed a biotinylated-G4C2 RNA pull down assay with nuclear lysates from HEK293T cells transiently expressing MATR3 (Fig. 1f). Consistent with previous reports, MATR3 wildtype was successfully pulled down with biotinylated (G4C2)_10_, indicating that there is physical interaction between G4C2 RNA and MATR3 (Fig. 1g). Interestingly, the interaction was diminished by RNA-binding deficient variant, MATR3-ΔRRM2, that has a truncation in the primary RNA-binding domain of the protein (Fig. 1g). This suggests that MATR3 binds to G4C2 RNA through its RNA-binding domain and is subsequently sequestered into pathogenic G4C2 foci found in patient-derived tissues and iPSC-MNs.

### MATR3 levels and subcellular localization altered in C9-ALS patient-derived iPSC-neurons

**Fig. 2:**
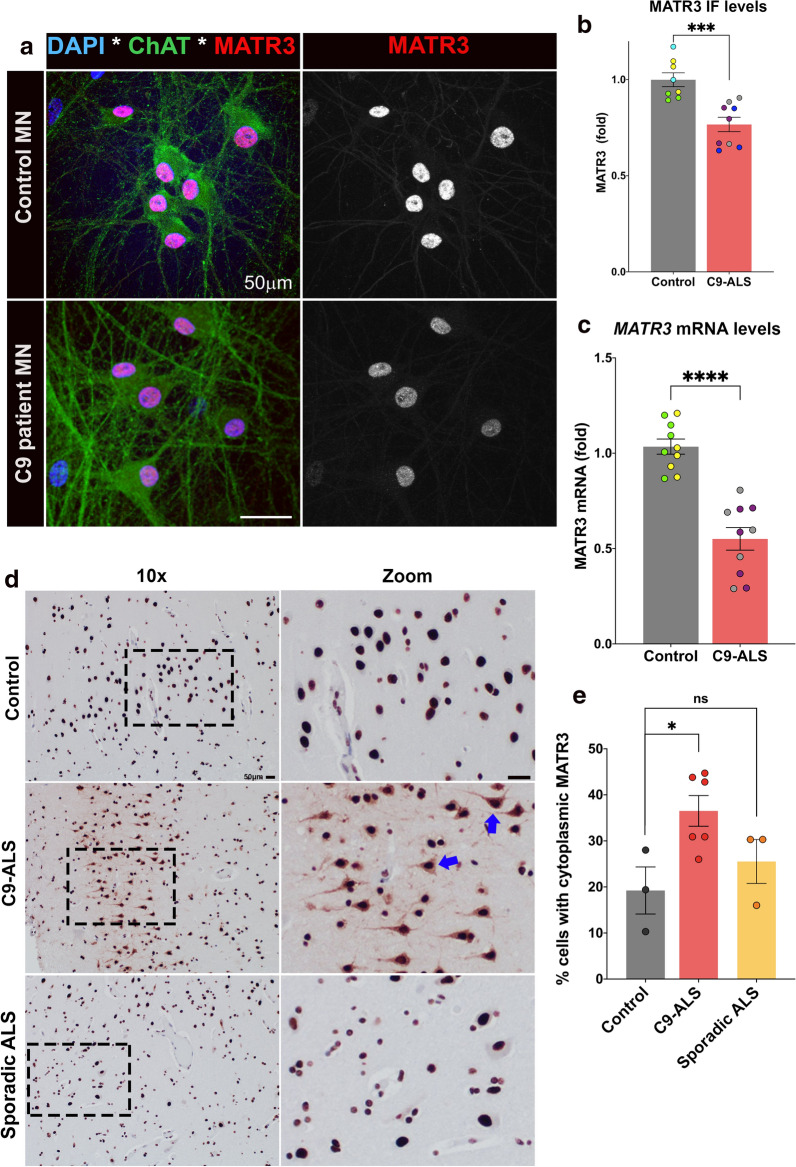
MATR3 levels and sub-cellular localization are perturbed in C9-ALS patient neurons and in post-mortem brain tissue. **a** Representative confocal images of control and C9-ALS patient-derived iPSCs that were differentiated to neurons (represented by ChAT^+^, green) and stained for endogenous MATR3 (red/gray). **b** Quantification of endogenous MATR3 immunofluorescence levels in ChAT^+^ neurons revealed significantly lower MATR3 levels in C9-ALS iPSC-MNs compared to that in control iPSC-MNs (Unpaired t-test) n = 2-3 independent differentiations from each independent control and C9-ALS iPSC-derived MN. iPSC-MNs from control: Ctrl #1 (yellow circle), Ctrl #2 (green circle), Ctrl #3 (cyan circle). iPSC-MNs from C9-ALS: C9-ALS #1 (gray circle), C9-ALS #2 (blue circle), C9-ALS #3 (purple circle). **c** Quantification of *MATR3* mRNA fold change in control and C9-ALS iPSC MNs showed significantly reduced *MATR3* mRNA levels in C9-ALS iPSC-MNs compared to that in control (Mann–Whitney U-test). n = 3 independent differentiations from each independent control and C9-ALS iPSC-derived MN. iPSC-MNs from control: Ctrl #1 (yellow circle), Ctrl #2 (green circle). iPSC-MNs from C9-ALS: C9-ALS #1 (gray circle), C9-ALS #3 (purple circle). **d** Representative IHC staining for MATR3 in entorhinal cortex sections from control, C9-ALS and sporadic (non-C9) ALS patient post-mortem brains. MATR3 was predominantly nuclear in control and sporadic ALS tissues, whereas in C9-ALS patient tissue, MATR3 also showed intense cytoplasmic signal (blue arrows). Scale = 50 µm. **e** Quantification of percentage of neurons with cytoplasmic MATR3 revealed increased percentage of neurons with cytoplasmic MATR3 staining in C9-ALS compared to control (Unpaired t-test). Control: 3 cases (n = 330; 135; 93); C9-ALS: 6 cases (n = 93; 485; 217; 310; 500; 251) and non-C9 sporadic-ALS: 3 cases (n = 316; 197; 402). Error bars indicate S.E.M. *p value < 0.05; ***p value < 0.001; ****p value < 0.0001

To investigate overall sub-cellular distribution of MATR3, we performed immunocytochemistry for endogenous MATR3 in C9-ALS iPSC-MNs (Fig. 2a). We observed a marked decrease in nuclear MATR3 immunoreactivity in the C9orf72 iPSC-MNs compared to that in control iPSC-MNs (Fig. 2a, b). Complementary to protein immunoreactivity levels, we also observed a concurrent decrease in *MATR3* mRNA levels in C9-ALS iPSC-MNs compared to controls (avg. fold change = 0.47 for C9 #1 and 0.43 for C9 #2) (Fig. 2c). To verify that this phenomenon is not a consequence of global change in levels of nuclear proteins, we immunostained C9-ALS patient iPSC-MNs for Histone H3 and found that C9-ALS iPSC MNs exhibited no significant difference in nuclear Histone H3 levels (Additional file 3: Figure S3A, B). Concurrently, we determined that the mRNA levels of *HIST1H3D*, which encodes one of the Histone H3 variants, and found that mRNA levels of Histone H3 were also not altered in C9-ALS iPSC MNs compared to control iPSC-MNs (Additional file 3: Figure S3C). Overall, our results from patient post-mortem brains and iPSC-MNs suggest that disruption in levels and localization of MATR3 is specific to C9-ALS.

Next, we used immunohistochemistry to determine if the subcellular localization of MATR3 is affected in C9-ALS patient post-mortem brain tissue. In control non-ALS cases, MATR3 was predominantly nuclear in entorhinal cortex sections, with sparse cytoplasmic signal detected in a small percentage of cells (Fig. 2d). On the other hand, examination of C9-ALS patient post-mortem brains revealed a significantly high percentage of cells with cytoplasmic immunoreactivity for MATR3 compared to that in control subjects (Fig. 2d, e). Interestingly, we found that in sporadic ALS patients, who don’t carry the hexanucleotide repeat expansion mutation in C9orf72, MATR3 staining in the cytoplasm more was comparable to that observed in control tissues (Fig. 2d, e). This suggests that increased cytoplasmic MATR3 pathology in post-mortem brains might be exclusive to C9-ALS.

### MATR3 is a strong suppressor of C9orf72 G4C2 HRE-mediated neurodegeneration in vivo

**Fig. 3:**
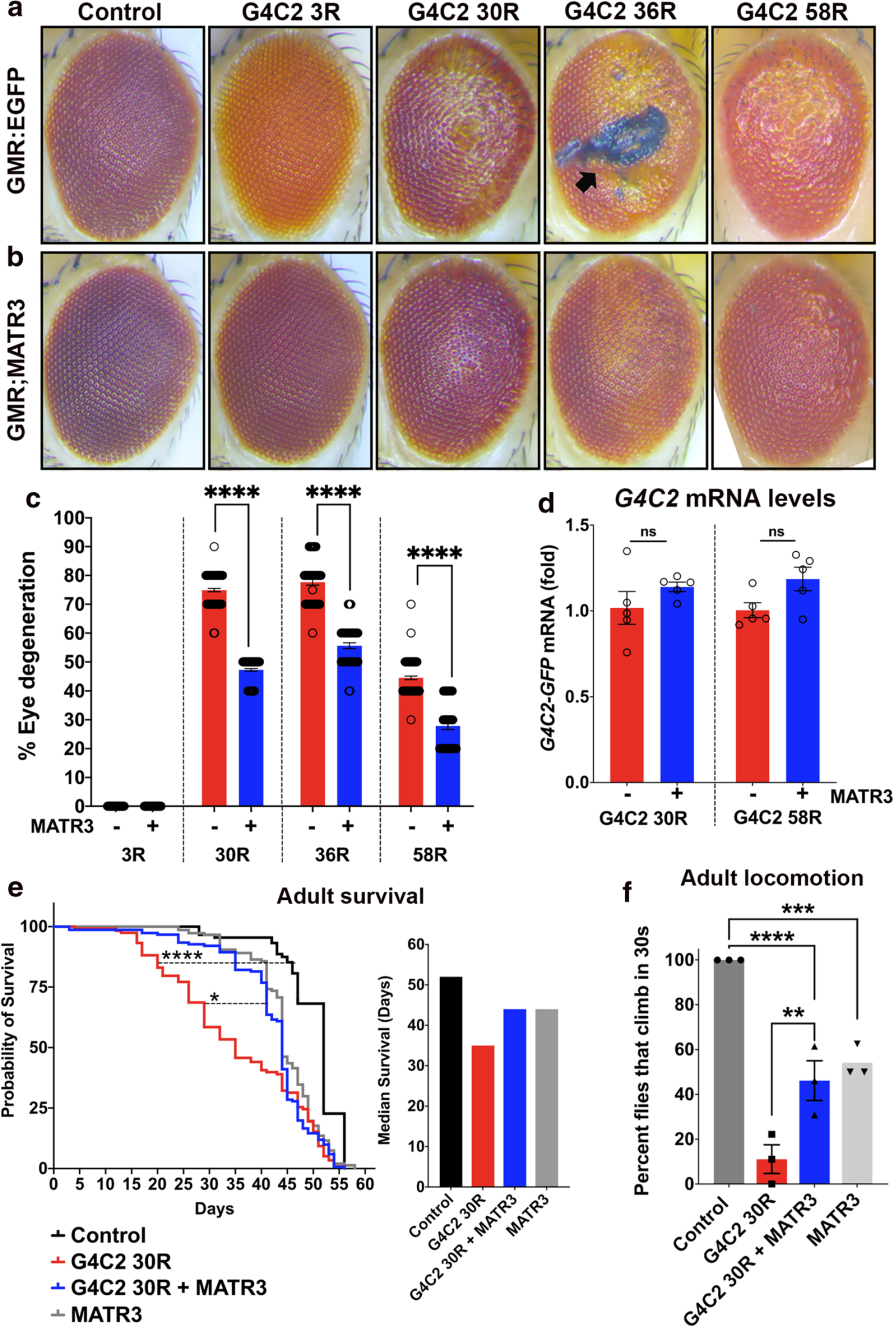
MATR3 is a strong modifier of C9orf72 G4C2-hexanucleotide repeat expansion (HRE)-mediated neurodegeneration in vivo. **a** Representative images of *Drosophila* eyes showing G4C2 hexanucleotide repeat expansion mediated eye degeneration in transgenic flies expressing 3 repeats (3R), 30 repeats (30R), 36 repeats (36R) and 58 repeats (58R) in the *Drosophila* eyes driven by GMR-gal4 driver. Flies expressing G4C2-3R had a comparable eye phenotype to control (GMR-GAL4; EGFP). Flies expressing G4C2-30R, 36R and 58R repeats exhibited signs of external eye degeneration including ommatidial fusion, bristle disorganization, depigmentation, and necrotic patches (arrow). These flies were expressing the G4C2 repeat expansion in the background of UAS-EGFP (control transgene) to account for GAL4 dilution. **b** Transgenic expression of human MATR3 in the G4C2 flies significantly ameliorated the external eye degenerative phenotypes and restored ommatidial structure and pigmentation. **c** Quantification of eye phenotypes showed statistically significant rescue in eye degeneration in G4C2-HRE flies upon expression of MATR3 (Kruskal–Wallis test). n ≥ 50 flies per genotype. **d** Quantification of mRNA levels of *G4C2*-*GFP* in heads from flies expressing G4C2-30R and 58R that are GFP-tagged, revealed no change in *G4C2*-*GFP* mRNA levels upon MATR3 expression (Kruskal–Wallis test). n = 5 per genotype. **e** Kaplan–Meier survival curve of flies conditionally expressing G4C2-30R in adult neurons, driven by ElavGS-GAL4 driver, showed significant reduction in longevity (left) and in median survival (right) (Log-Rank Mantel Cox test). n = 100 flies per genotype. **f** Neuronal expression of G4C2-30R caused profound motor dysfunction in adults. Quantification of the percentage of flies that can climb in 30 s indicated severe locomotion defects in G4C2 30R-expressing flies. Expression of MATR3 partially rescued the motor defects (One-way ANOVA). n ≥ 30 flies per genotype. Error bars indicate S.E.M. *p value < 0.05; **p value < 0.01; ***p value < 0.001; ****p value < 0.0001

Our results so far indicated that G4C2-HRE in C9orf72 not only physically associates with MATR3, but also significantly impacts the levels and sub-cellular localization of MATR3. So, we sought to determine whether MATR3 and G4C2 HRE genetically interact in vivo in *Drosophila*. We utilized previously published *Drosophila* models of G4C2-HRE toxicity, particularly transgenic models expressing 3 repeats (3R), 30 repeats (30R), 36 repeats (36R) and 58 repeats (58R) of G4C2 [35–37]. When expressed in the eye, these models exhibit varying degrees of eye degeneration characterized by depigmentation, ommatidial fusion and/or loss, bristle disorganization, and in the case of 36R, necrotic patches (Fig. 3a). We found that ectopic expression of human transgenic MATR3 wildtype in these fly eyes significantly ameliorated repeat-induced eye degeneration, including amelioration of the necrotic patches caused by 36R (Fig. 3b). Phenotypic quantification of eye degeneration showed statistically significant suppression of toxicity across all G4C2-HRE models upon expression of MATR3 (Fig. 3c). Additionally, we observed that while G4C2 30R-mediated eye degeneration is exacerbated with aging to 30-days, expression of MATR3 in these flies continued to suppress the degeneration, even at later ages (Additional file 4: Figure S4A,C). Using RT-qPCR, we determined that the mRNA levels of G4C2 are not significantly changed upon expression of MATR3 (Fig. 3d), indicating that the suppression in eye phenotype is most likely not due to downstream effects of G4C2 expression.

To determine if ectopic expression of MATR3 in G4C2-HRE flies can suppress motor deficit, we assessed locomotion ability in adult flies expressing G4C2-30R in neurons. Expression of G4C2-30R alone results in severe motor degeneration in these animals, with only ~ 10% flies able to climb at all (Fig. 3f). Ectopic expression of MATR3 in these flies significantly improved this motor function deficit (Fig. 3f). Additionally, conditional expression of 30R in neurons markedly reduced survival in these flies, a phenotype which was also rescued by MATR3 expression (Fig. 3e). Owing to functional and structural similarity of MATR3 to other ALS-associated RNA-binding proteins with known RNA-recognition motifs, including TDP43, FUS and EWSR1, we asked if the eye degeneration of G4C2-30R could be suppressed by ectopic expression of TDP43, FUS and EWSR1. We found that expression of neither of these RBPs suppressed the eye degeneration in G4C2-30R flies (Additional file 5: Figure S5A, B). In fact, due to intrinsic eye degeneration that is induced by ectopic expression of these RBPs, particularly in the case of FUS and EWSR1, the eye degeneration compounded in the presence of G4C2-30R (Additional file 5: Figure S5A, B). Interestingly, both FUS and EWSR1 have been identified as binding partners of G4C2 RNA, suggesting that despite being a part of the shared G4C2-RBP proteome, MATR3 suppresses G4C2-HRE neurodegeneration through distinct mechanisms.

### RNA-binding domain of MATR3 required to suppress G4C2 toxicity in vivo

**Fig. 4:**
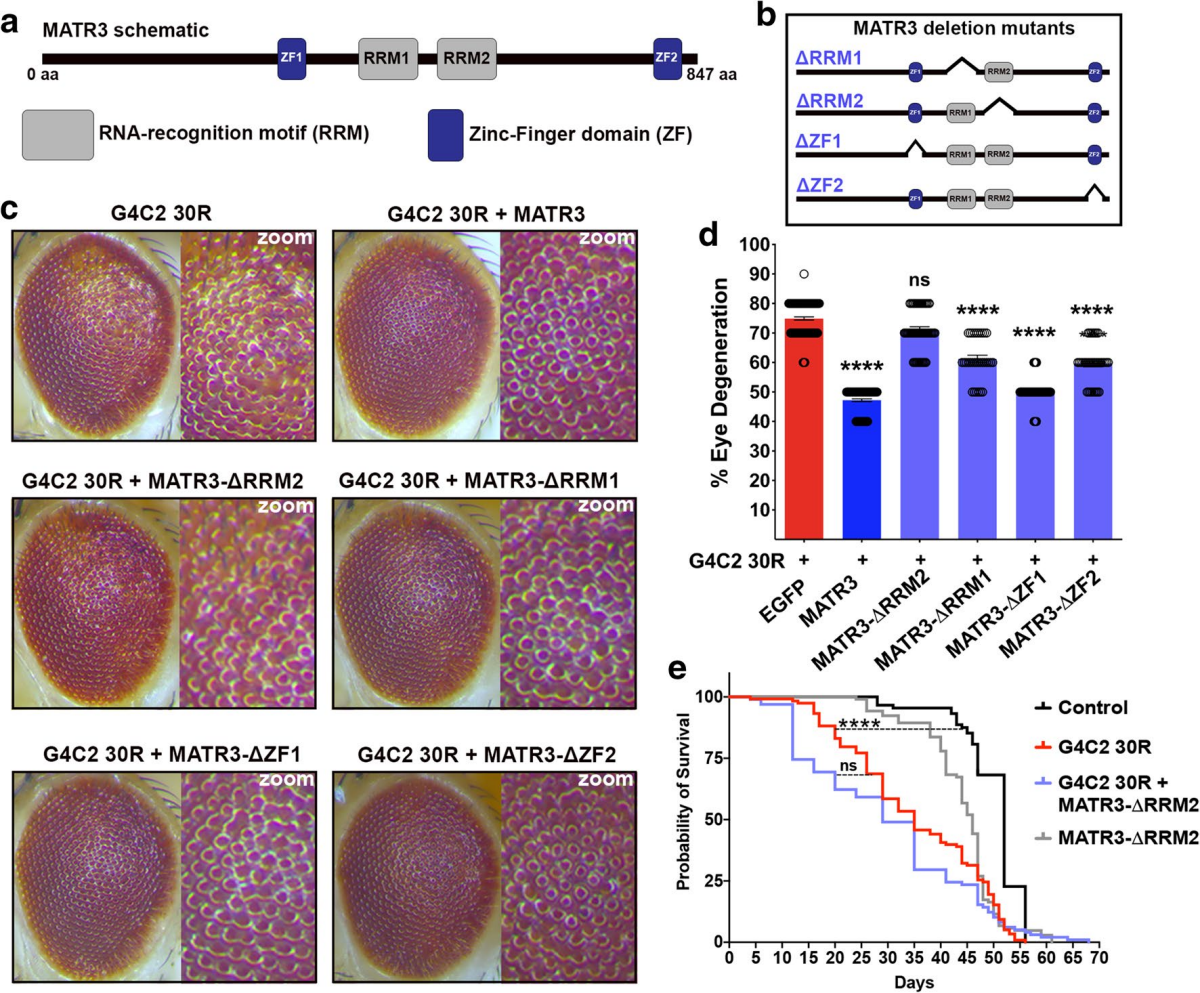
RRM2 domain of MATR3 required to mediate G4C2-HRE toxicity in vivo. **a** Schematic of 847 amino acid (aa)-long MATR3 protein and its functional domains: two RNA-recognition motifs (RRM1/2) and two zinc-finger domains (ZF1/2). **b** Schematic of MATR3 with each functional domain deleted to generate deletion mutants: ΔRRM1, ΔRRM2, ΔZF1, ΔZF2. **c** Representative images of *Drosophila* eyes from flies co-expressing G4C2-30R with MATR3 (full-length) or deletion variants (ΔRRM1, ΔRRM2, ΔZF1, ΔZF2). Zoom panels emphasize the degree of ommatidial disorganization and depigmentation. MATR3-ΔRRM2 suppresses G4C2-30R toxicity to a lesser degree compared to MATR3 wildtype, indicated by increased degenerative phenotypes including de-pigmentation and ommatidial fusion compared to G4C2-30R + MATR3 (full-length). **d** Quantification of eye degeneration revealed no difference in G4C2-30R + MATR3-ΔRRM2 compared to G4C2-30R alone (Kruskal–Wallis test) n ≥ 50 per genotype **e** Kaplan–Meier survival curve of adult flies showed that neuronal expression of MATR3-ΔRRM2 mutant in G4C2-30R flies did not modify reduced survival defect in G4C2-30R flies (Log Rank Mantel Cox test) n = 100 flies per genotype. Error bars indicate S.E.M. *p value < 0.05, ****p value < 0.001

We next sought to investigate which potential function of MATR3 is mediating suppression of G4C2-HRE toxicity. MATR3 has four known functional domains: two zinc finger motifs (ZF1 and ZF2), known to have DNA-binding and putative RNA-binding properties [29, 38], and two tandem RNA recognition motifs (RRM1 and RRM2), with RRM2 domain demonstrated to exhibit more predominant RNA-binding activity (Fig. 4a) [26]. To map the genetic interaction between G4C2 and MATR3 to the functional domains of the protein, we generated transgenic lines with each of the four functional domains deleted: ΔRRM1, ΔRRM2, ΔZF1 and ΔZF2 (Fig. 4b, Additional file 6: Figure S6A). MATR3 with truncation in either zinc-finger domains (ΔZF1 and ΔZF2) still retained its suppressive effect on G4C2-30R eye degeneration (Fig. 4c, d). On the other hand, deletion of RRM domains, particularly the RRM2 domain, had a detrimental effect in the ability of MATR3 to suppress G4C2-30R toxicity (Fig. 4c, d). This was even more evident in aged flies, where at day-30, expression of MATR3-ΔRRM2 was effective in suppressing G4C2 30R-mediated eye degeneration to the same extent as MATR3 (Additional file 4: Figure S4B). Expression of MATR3 deletion mutants alone in *Drosophila* eyes did not cause any external eye degeneration (Additional file 6: Figure S6B, Additional file 4: Figure S4B), confirming that the phenotypes observed in flies co-expressing G4C2-30R and MATR3-ΔRRM2 is not due to cumulative toxicities. Consistent with the eye phenotypes, in flies conditionally expressing G4C2-30R in adult neurons, ectopic expression of MATR3-ΔRRM2 was also unsuccessful in increasing their longevity (Fig. 4e). This suggests that the RNA-binding domain of MATR3 is required to suppress G4C2 HRE toxicity in vivo.

### Ectopic expression of MATR3 suppresses RAN translation in mammalian cells

**Fig. 5:**
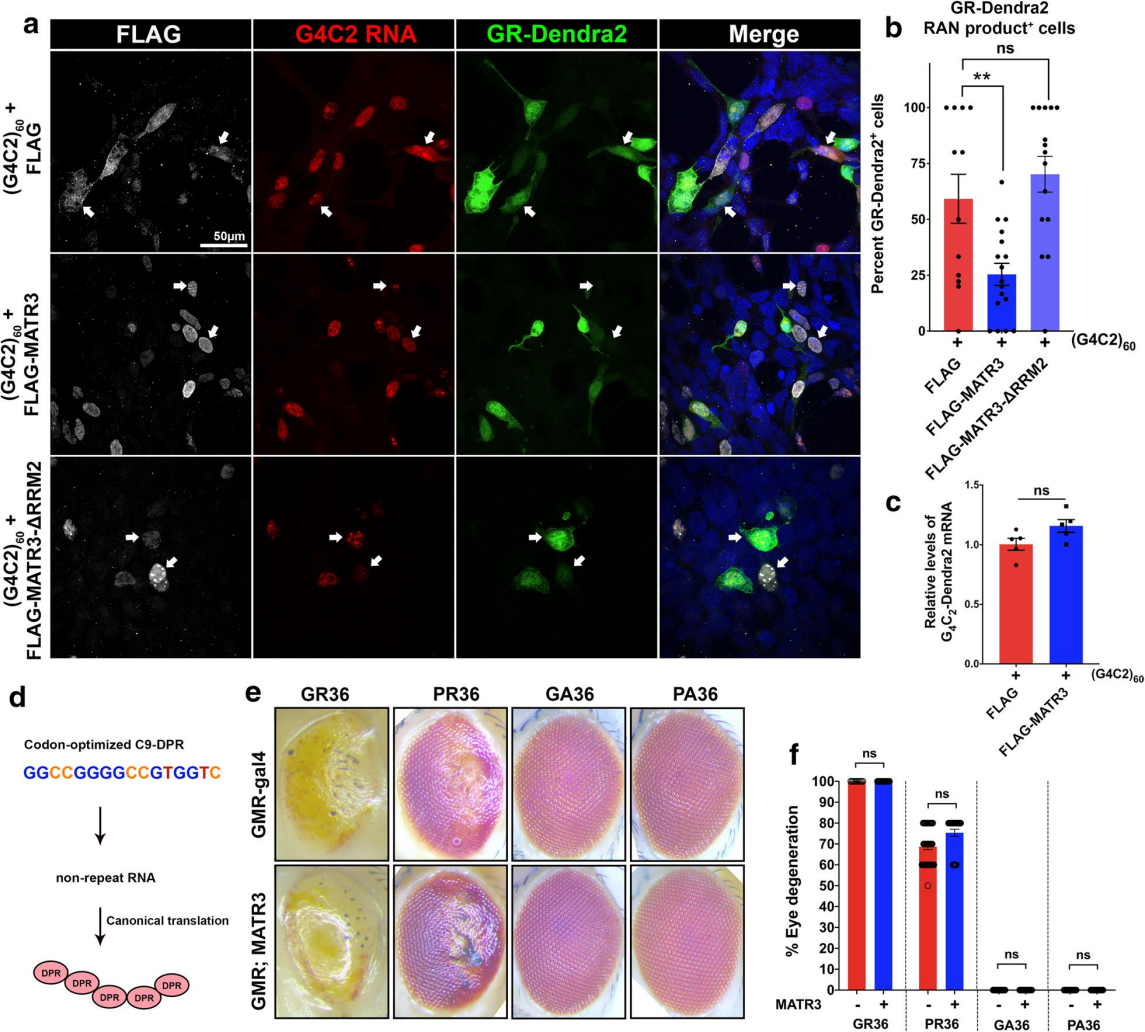
MATR3 overexpression mitigates DPR production from G4C2_60_ transcripts in an RNA-dependent manner. **a** Representative confocal images of HEK293T cells co-transfected with G4C2_60_-Dendra2 RAN translation reporter plasmid and either FLAG (empty vector), FLAG-MATR3 or FLAG-MATR3-ΔRRM2 plasmids. G4C2_60_ expression resulted in formation of G4C2 RNA foci (red) and, further, the G4C2_60_ transcripts also undergo RAN translation in a subset of cells to produce dipeptide repeats (DPRs), indicated by Dendra2 signal (green) that is in frame with polyGR (white arrows). **b** Quantification of the percentage of cells that produce GR-Dendra2 (green) RAN product showed that overexpression of FLAG-MATR3 (gray) significantly reduced RAN translation of G4C2_60_ transcript to GR-Dendra2 RAN product. Overexpression of FLAG-MATR3-ΔRRM2 did not impact GR-Dendra2 production in these cells (One-way ANOVA) n = 15-18 per group. **c** Quantification of *G4C2*_*60*_-*Dendra2* mRNA levels in cells expressing FLAG (empty vector) or FLAG-MATR3 showed no significant difference (One-way ANOVA) n = 5 per group. **d** Schematic of previously-published codon-optimized approach to produce dipeptide protein repeats by canonical translation of non-repeat RNA (i.e. not carrying GGGGCC repeats). **e** Representative images of *Drosophila* eyes and **f** quantification of external eye degeneration from flies expressing codon-optimized DPRs: GR36, PR36, GA36 and GP36. Flies expressing PR36 and GR36 developed significant external eye degeneration that is more severe is GR36 compared to PR36. Flies expressing either GA36 or GP36 did not show any external eye degeneration. Expression of MATR3 in these flies did not modify the phenotypes (Kruskal–Wallis test) n ≥ 50 per genotype. Error bars indicate S.E.M. **p value < 0.01

G4C2-HRE transcript potentially induces neurodegeneration through formation of nuclear RNA foci and through RAN-translation of the G4C2 RNA into toxic DPRs that form neuronal inclusions [6, 39]. To investigate which of these pathogenic mechanisms is potentially altered by MATR3 expression, we used a reporter G4C2 construct, consisting of ~ 60 repeats (termed G4C2_60_), with a fluorescent Dendra2 reporter in the GR-frame which is only detected if the transcript undergoes RAN translation. We co-transfected HEK293T cells with the G4C2_60_-Dendra2 reporter plasmid along with either FLAG or FLAG-MATR3 plasmid. Using RNA-FISH combined with ICC, we confirmed that overexpression of the FLAG-empty vector and G4C2_60_-Dendra2 reporter in HEK293T cells results in formation of G4C2 RNA foci in the nucleus, and importantly, that a subset of these cells (~ 59%) produced GR-Dendra2 RAN-translated product (Fig. 5a). We observed that overexpression of FLAG-MATR3 with G4C2_60_-Dendra2 significantly decreased in the percentage of G4C2-expressing cells that produced GR-Dendra2 RAN product (by ~ 58%) (Fig. 5a, b). Ectopic expression of MATR3 had no impact on the levels of G4C2_60_-Dendra2 transcripts (Fig. 5c), indicating that the apparent reduction in RAN-translated products upon MATR3 overexpression is possibly through post-transcriptional mechanisms.

Given that RRM2-deficient MATR3 was not able to suppress G4C2 toxicity in vivo, we investigated if RRM2 deletion results in a similar outcome in vitro. We found that overexpression of FLAG-MATR3-ΔRRM2 with G4C2_60_-Dendra2 did not alter the percentage of G4C2-expressing cells that produced GR-Dendra2 (Fig. 5a, b). We also sought to test if MATR3 could suppress G4C2 toxicity through any mechanisms independent of G4C2 repeat-bearing RNA. To test this in vivo, we utilized codon-optimized dipeptide repeat (DPR) fly lines [37, 40] that produce the end-product toxic peptides, however, bypassing the production of transcripts that carry the G4C2 repeat RNA (Fig. 5d). The codon-optimized expression of GR or PR dipeptide repeats (GR36 and PR36) in fly eyes results in severe degenerative phenotype (Fig. 5e, f). We observed that ectopic expression of MATR3 in these flies did not alleviate the degenerative phenotype (Fig. 5e, f). Additionally, codon-optimized expression of GA and PA dipeptide repeats (GA36 and PA36) does not result in any external eye degeneration in flies with or without MATR3 (Fig. 5e, f). This suggests that ectopic expression of MATR3 does not impact post-translational toxicity associated with C9-DPRs.

### MATR3 modulates G4C2-HRE RNA foci in C9-ALS patient neurons

**Fig. 6:**
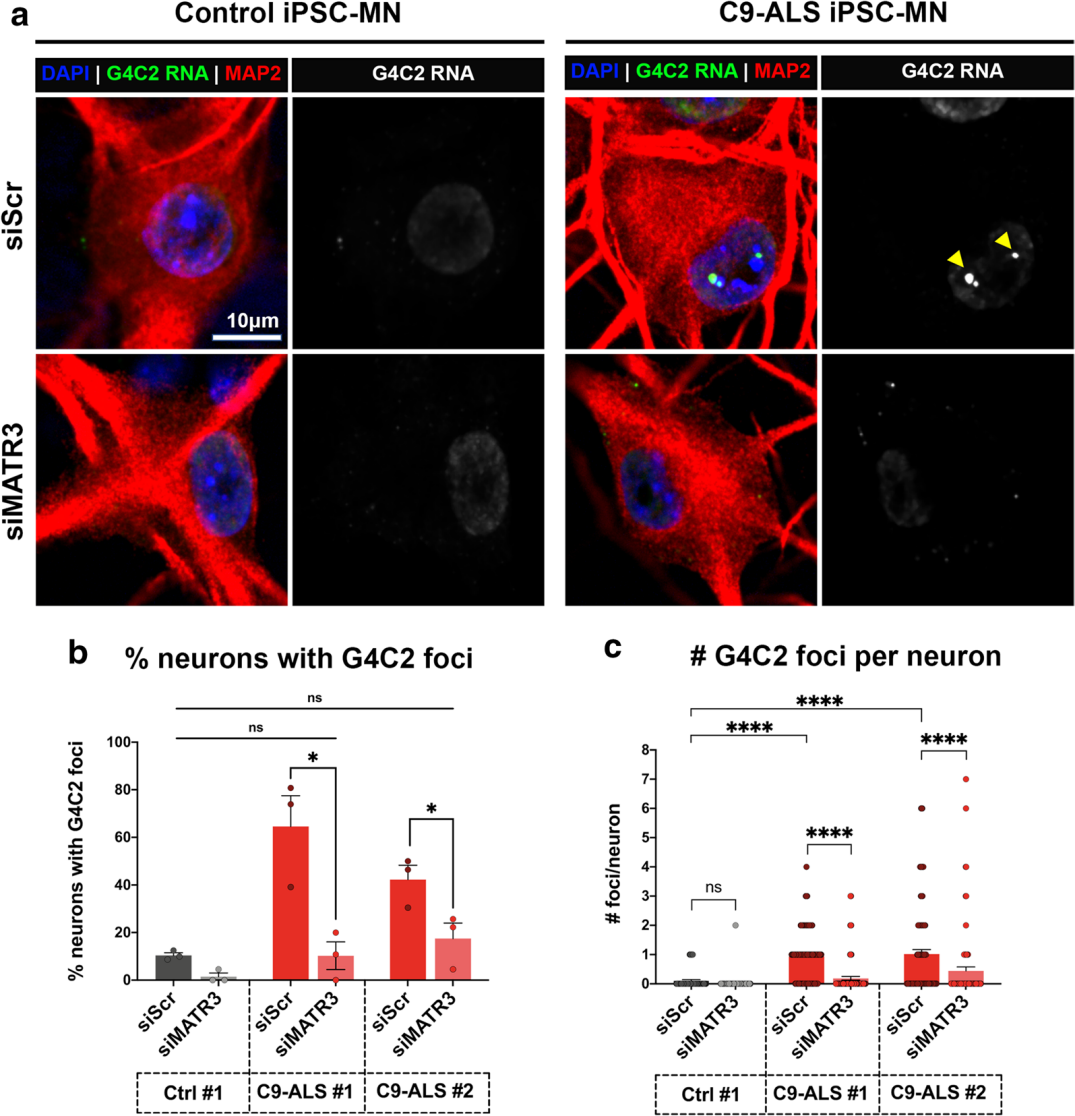
G4C2 RNA foci modulated by MATR3 in C9-ALS patient iPSC-MNs. **a** Representative confocal images of control (left) and C9-ALS patient (right) iPSC-MNs, indicated by MAP2 (red), transfected with scrambled siRNA or *MATR3* siRNAs. FISH-IF was performed to stain nuclear G4C2 RNA foci (green; yellow arrowheads) in these neurons. **b**, **c** Quantification of the percentage of neurons that are G4C2 foci-positive (**b**), and number of G4C2 foci per neuron (**c**) in iPSC-MNs from one control (Ctrl #1) and two independent C9-ALS (C9-ALS #1, C9-ALS #2). Knocking down MATR3 significantly reduced the percentage of neurons that form G4C2 foci (**b**) and also reduced number of G4C2 foci per neuron (**c**) compared to scrambled-control in both C9-ALS iPSC-MNs (Kruskal–Wallis test). n = 24-50 neurons Each data point (circle) represents data from neurons pooled from 3 independent differentiations. Error bars indicate S.E.M. ****p value < 0.0001

Collectively, our data suggests a G4C2 expanded repeat RNA-dependent genetic interaction between MATR3 and C9-ALS. To further validate this interaction in the context of C9-ALS disease neurons, we utilized siRNA (siMATR3) to significantly knockdown endogenous MATR3 in C9-ALS iPSC MNs (Additional file 7: Figure S7A, B). We observed that knocking down endogenous MATR3 resulted in a significant decrease in the percentage of neurons that exhibit G4C2 foci (Fig. 6b), and simultaneously, the number of G4C2 foci per individual neuron (Fig. 6a–c), consistently across two independent patient-derived C9-ALS iPSC MNs (Fig. 6b), confirming a disease-specific genetic interaction between MATR3 and G4C2 repeat RNA in these neurons.

## Discussion

GGGGCC hexanucleotide repeat expansion in the first intron of C9orf72 gene leads to neurodegeneration through a combination of loss-of-function of endogenous C9orf72 protein, and gain-of-function of toxic repeat-associated RNA and protein aggregates [41]. The gain-of-function RNA toxicity, that arises from bidirectional transcription of the repeats, can manifest in multiple ways. G4C2 and C4G2 repeat RNA form pathologic RNA aggregates, or RNA foci, within the brain and spinal cord of C9-ALS/FTD patients [11, 12, 42–45]. These RNA foci directly interact with and sequester essential RNA-binding proteins and thereby compromise their normal function [23].

Colocalization and G4C2-pull down studies in C9-ALS brain tissues as well as iPSC-derived neurons have identified multiple RNA-binding proteins (RBPs) that interact with the G4C2 repeat RNA, including hnRNP-family proteins (hnRNPA1, A2/B1, A3, K, H1, H3), ALYREF, ADARB2, RanGAP1 and MATR3 [7, 24, 25]. Meta-analysis of all proteins that make up the G4C2-RBP interactome has identified two independent groups that determined MATR3 to be one of the interactors of G4C2 RNA [7, 24, 25]. Consistent with these reports, we found that MATR3 binds to G4C2 RNA, mediated partially by its RRM2 RNA-binding domain. However, the disease relevance of this interaction was previously unknown. In this study, we identify for the first time colocalization between G4C2 RNA foci and MATR3 in C9-ALS/FTD patient-derived brain tissue and iPSC-neurons, thus linking MATR3 to C9-ALS/FTD disease pathogenesis. RBPs have been shown to have differential binding affinities to G4C2 RNA depending on its higher-order secondary structure. For example, hnRNPH binds to both G-quadruplex and hairpin conformations of G4C2 [24], and on the other hand, SRSF1, nucleolin and RanGAP1 exhibit higher binding affinity to G-quadruplex [24, 46]. Elucidation of differential binding affinities of MATR3 to G4C2-secondary structures are required to further characterize the nature this interaction. Interestingly, we also found that truncation of the RNA-binding domain of MATR3 does not completely abolish the interaction between MATR3 and G4C2 RNA, suggesting that there might be other factors mediating the interaction. In fact, a comparison of the protein interactome of MATR3 [47–49] and G4C2 RNA [7, 24, 25] reveals multiple common RNA-binding proteins including members of the hnRNP and DDX family of proteins as well as components of nuclear bodies including SFPQ and NONO. Thus, MATR3 could simultaneously be binding to G4C2 repeats directly and associate with other RNA-binding proteins that bind to G4C2, that multimerize to form the G4C2 foci. The results described in this study suggests protective effects of MATR3 in C9orf72-ALS by restoring RBP homeostasis.

Our data from post-mortem human brain tissues also indicate that sub-cellular localization of MATR3 is perturbed in C9-ALS neurons. MATR3 is a predominantly nuclear protein under physiological conditions, whereas, in C9-ALS patients, MATR3 exhibits significant cytoplasmic mislocalization. Disruption in MATR3 subcellular localization has previously been reported in other forms of ALS cases, including in C9-ALS and sporadic-ALS patient brain tissues [30, 34]. However, contrary to previous observations in sporadic ALS patient tissues [34], we found that MATR3 localization in sporadic ALS brain tissues were comparable to that in control brains. The discrepancy between our observations could be potentially due to differences in patient demography (Caucasian patients in this study vs. Japanese patients in Tada et al. [34]) and difference in the age of death (avg. 58 years in this study vs. avg. 70 years in Tada et al. [34]). Additionally, the MATR3 antibodies used by both studies each recognize different epitopes which could also possibly explain the differences in our observations. In addition to perturbed sub-cellular localization, the levels of MATR3 are significantly reduced in C9-ALS neurons, interestingly at the transcriptomic level. Like many other RBPs, MATR3 is known to autoregulate its own levels [26, 27, 50]. Thus, reduced MATR3 levels in C9-ALS neurons could be a direct consequence of sequestration of MATR3 into G4C2 RNA foci. This raises the possibility that MATR3 sequestration in G4C2 foci might mimic a potential loss-of-function of endogenous MATR3, resulting in disruption of its native functions. Initially discovered as a component of the nuclear matrix, MATR3 is best known for its role in RNA metabolism, including in alternative splicing, mRNA stability and mRNA export [26–28, 48, 51, 52]. In fact, knocking down MATR3 has been reported to lead to transcriptome-wide changes in mRNA levels as well as alternative splicing events in mammalian cells [26, 27]. In C9-ALS, widespread alterations of alternative splicing and alternative polyadenylation site usage has been identified in cerebellar tissues from patients [53]. Thus, depleted levels of MATR3 in C9-ALS patient neurons might be one of the mechanistic links to wide-scale splicing defects that contributes to neurodegeneration.

In addition to linking MATR3 with neuropathology in C9-ALS patient-derived neurons, we identified MATR3 as a strong suppressor of G4C2-HRE toxicity in vivo. Specifically, we found that deletion of the RNA-binding domain of MATR3 (MATR3-ΔRRM2) significantly reduced the ability of MATR3 to suppress G4C2 toxicity in vivo. Importantly, our data is consistent with a model of RNA-mediated suppression of G4C2 toxicity. Furthermore, utilizing a reporter for RAN translation, we showed that overexpression of MATR3 suppresses DPR production in human cells dependent on the RRM2 domain, suggesting that MATR3 interaction with G4C2 RNA is important for suppressing the C9-DPR toxicity. However, a caveat is that the cells overexpressing MATR3-ΔRRM2 also have endogenous wildtype MATR3 with, presumably, a fully functional RNA-binding domain. Thus, the true effects of RRM2 deletion upon DPR production would have to be further investigated in the context of MATR3 knockout or point mutations in the RNA-binding domain of endogenous MATR3 that debilitate its RNA-binding function. Nevertheless, in *Drosophila* models that produce DPR products through canonical translation of codon-optimized transcripts that don’t bear the G4C2 repeats, MATR3 expression had no effect on the polyGR- or polyPR-mediated toxicities. This suggests that overexpression of MATR3 has no impact on post-translational toxicities conferred by DPR products in C9orf72-ALS, and that it is likely acting through upstream mechanisms which might be dependent on its RNA-binding function. Interestingly, we also demonstrated that in patient-derived neurons, modulating MATR3 levels directly impacts G4C2 RNA foci formation, raising a possibility that RNA foci and RAN translation/DPR mechanisms are linked through MATR3.

Deriving from the patient pathology showing physical- and colocalization-interaction between MATR3 and G4C2 RNA, and genetic interaction between MATR3 and G4C2, both in vivo and in C9-ALS patient-derived neurons, we suggest two possible ways in which this interaction could be involved in C9-ALS pathobiology: (1) Decreased levels of nuclear MATR3 and/or cytoplasmic localization of MATR3 that could perturb its physiological functions due to alteration in the subcellular distribution; and, (2) nuclear sequestration of G4C2 RNA foci and suppression of RAN translation. It is possible that loss of nuclear MATR3 and/or cytoplasmic localization of MATR3 in C9-ALS neurons could be promoting RAN translation of G4C2-HRE repeat RNA into toxic DPRs. We speculate that in cells expressing sufficient MATR3 or overexpressing MATR3, it interacts with G4C2 RNA to sequester the expanded repeat RNA within the nucleus, and inversely, decreases availability of G4C2-HRE RNA for RAN-translation. Therefore, MATR3 could be imparting a neuroprotective role to G4C2 RNA foci in C9-ALS. MATR3 may possibly aid in G4C2 RNA foci formation through mediating post-transcriptional modifications that increase the stability of G4C2 higher-order secondary structures and thus, serving to stabilize the foci. Our data also raises the possibility that MATR3 could be inhibiting nuclear export of G4C2 repeat RNA to the cytoplasm and this, in turn, reduces the production of RAN proteins. The benefit of inhibiting nuclear export of repeat expansion RNA has been previously explored in C9-ALS [39, 54], as well as in other microsatellite expansion-mediated neurological disorders, including FXTAS [55], SCA31 [56] and myotonic dystrophy 2 [57]. Unbiased genetic screens in *Drosophila* models of C9-ALS, including ones used in this study, have identified that depletion of nuclear export adapters, such as ALYREF and SRFS1, is protective in these models [54, 58, 59]. Indeed, in patient-derived neurons, SRSF1 has been demonstrated to facilitate export of G4C2-HRE RNA, and furthermore, depletion of SRFS1 leads to an increase in the nuclear retention of G4C2 RNA, and concomitantly, a decrease in the production of RAN-translated toxic DPRs [54]. It is likely that MATR3 could be acting through the same or similar pathways to promote nuclear retention of G4C2-HRE RNA and subsequent sequestration into foci. Further investigation into the mechanisms that link MATR3-G4C2 RNA binding and culminating in RAN translation is needed to fully elucidate the potential of restoring normal levels of MATR3 and/or overexpression of MATR3 in suppressing C9-ALS neurodegeneration.

## Materials and methods

### Human post-mortem brain tissues

The patients and controls were sampled from a similar age range. At the time of death, the age of control individuals (n = 3) ranged between 54 and 76 years, C9-ALS patients (n = 6) ranged between 51 and 67 years and that of non-C9 ALS patients (n = 3) ranged between 46 and 58 years. Clinical and neuropathological information of C9-ALS, non-C9 ALS and age-matched non-neurological control post-mortem human brain tissues is listed in Table 1.

**Table 1:** Summary of clinical and neuropathological features of control (healthy), C9-ALS and sporadic (non-C9) ALS patient post-mortem brain tissues used in this study

Case ID	Gender	Race	Age at death	Age at onset	Duration of disease after diagnosis (years)	Primary onset	Clinical diagnosis	Primary neuropathology diagnosis	Additional neuropathology diagnoses
CW01-97	F	W	57	N/A	N/A	N/A	Normal*	Metastatic lung carcinoma	1. Primary age-related Tauopathy; 2. Mild acute ischemic injury, hippocampus
CW01-98	M	W	76	N/A	N/A	N/A	Normal*	Alzheimer’s disease neuropathologic change, intermediate level	Remote frontal lobe infarct
CW99-100	M	W	54	N/A	N/A	N/A	Normal*	No pathologic changes	
CW03-130	M	W		Not available	Not available	Not available	ALS	ALS (C9orf72 pattern)	
CW07-94	F	W	63	59	4	Hand weakness	ALS	ALS (C9orf72 pattern)	Alzheimer’s disease neuropathologic change, low level
CW07-96	M	W	67	Not available	Not available	Not available	ALS	ALS (C9orf72 pattern)	Alzheimer’s disease neuropathologic change, low level
CW13-97	F	W	62	58	4	Leg weakness	ALS	ALS and FTLD-TDP (C9orf72 pattern)	Alzheimer’s disease neuropathologic change, low level
CW98-96	M	W	43	42	1	Not available	ALS	ALS (C9orf72 pattern)	
CW98-97	F	W	51	49	2	Not available	ALS	ALS (C9orf72 pattern)	
CW12-96		Unknown	59	57	1.7	Leg weakness	ALS	ALS	Alzheimer’s disease neuropathologic change, low level
CW15-99	F	W	51	46	5	Leg weakness	ALS	ALS	Alzheimer’s disease neuropathologic change, low level
CW12-97	M	W	58	58	0.6	Dysarthria	ALS	ALS	Primary age-related tauopathy

*M* male, *F* female, *W* white, *N/A* not applicable

*Negative for dementia or movement disorders

### Immunohistochemistry

Formalin-fixed, paraffin-embedded human entorhinal cortex sections of ALS/FTD subjects were retrieved from the Neurodegenerative Brain Bank at the University of Pittsburgh, following protocols approved by the University of Pittsburgh Committee for Oversight of Research and Clinical Training Involving Decedents (CORID).

Six µm thick sections were deparaffinized in xylene and rehydrated through graded ethanol (100%, 95%), followed by a 30-min incubation in methanol/hydrogen peroxide. Antigen retrieval was performed with citrate buffer, pH 6.0, for 20 min at 95 °C, followed by rinses with water and PBS/Tween. Sections were then blocked for 6 min at room temperature with Power Block (BioGenex, HK085-5K). Sections were incubated in primary antibody (rabbit anti-MATR3, 1:200; Abcam 151714) diluted in Antibody Diluent (ScyTek) for 45 min at room temperature, rinsed in PBS and then incubated with biotinylated anti-rabbit secondary antibody (Vector Laboratories, BA-1000, 1:200) for 30 min, Slides were then immersed in ABC solution for 30 min (ABC Elite, Vector Laboratories, PK6100) and visualized with Nova Red (Vector Laboratories, #SK-4800). After counterstaining with Mayer’s hematoxylin (Sigma, MHS32), slides were dipped in 1% Lithium carbonate bluing solution (Fisher, L119-500), dehydrated in a series of ethanols, cleared in xylene, and mounted with Permount mounting medium (Fisher, SP15-500).

#### Quantification of cytoplasmic MATR3 staining

Quantification of the MATR3 IHC immunostaining was performed in a blinded fashion. Bright-field images from multiple fields of the entorhinal cortex were used for analysis. Briefly, cells with MATR3 signal (brown/DAB) in the cytoplasm were counted and plotted as a percentage of the total number of cells in the field.

### Patient brain tissue RNA FISH and immunohistochemistry

Human control and ALS motor cortex sections (formalin-fixed, paraffin-embedded) were then permeabilized with 0.4% Triton X-100 for 15 min at room temperature following two 1× PBS washes and subsequently equilibrated in 2× SSC for 10 min at room temperature. Sections were then incubated in 50% formamide solution (in 1× SSC) for 10 min at 85 °C while 5′ TYE 563-labeled LNA probes (5TYE563/CCCCGGCCCCGGCCC, Exiqon, Batch # 620253) were incubated at 95 °C for 5 min. Probe hybridization was then performed by first adding 200 µL probe solution (300 µg/mL salmon sperm, 300 µg/mL E.Coli tRNA, 1.5 ng/uL probe in 100% formamide) and 200 uL hybridization buffer (20% dextran sulfate in PBS, 4 mg/mL BSA, 2× SSC, 2 mM RVC, 0.1× PBS) per section and incubation for 1 h at 66 °C. Sections were then washed first in 50% formamide (in 2× SSC) at 80 °C for 15 min prior to three 5-min washes in 2× SSC at room temperature, one 5-min wash in Tris–glycine solution (200 mM Tris pH 7.4, 7.5 mg/mL glycine) at room temperature, and one 5-min wash in TBS-50 (50 mM Tris pH 7.4, 150 mM NaCl) at room temperature. Following UV crosslinking, sections were blocked overnight at 4 °C (0.1% Triton X-100, 2% heat shock BSA in TBS-50). The next day, sections were incubated with primary antibodies (rabbit anti-Matrin-3, Abcam: ab84422, 1:500; mouse anti-NeuN, Abcam: ab104224, 1:500) in IF buffer (0.5% Protease-free BSA Fraction V (Roche), 0.5% Heat-shocked BSA Fraction V (Roche), 0.1% Triton X-100 in TBS-50) overnight at 4 °C. Sections were then exposed to four 5-min washes in IF buffer at room temperature prior to incubation with secondary antibodies (AlexaFluor 488/AlexaFlour 647, 1:800) for 1 h at room temperature. Four 5-min washes in IF buffer, two 5-min washes in TBS-50, two 5-min washes in 1× PBS + 2 mM MgCl_2_, and one 5-min wash in 1× PBS were then performed prior to mounting slides with Prolong Antifade Mounting Media with DAPI (Invitrogen).

#### Quantification of G4C2 FISH and MATR3 colocalization

For quantification of G4C2 RNA Foci and MATR3 colocalization in human tissue, Z stack images of 12–20 nuclei per patient from three C9 ALS/FTD patient motor cortex embedded paraffin sections were acquired using a Nikon A1R confocal microscope. Images were then deconvolved using 10 Landweber iterations on the Nikon element program. The single middle plane of the reconstructed RNA foci was then selected and analyzed for Matrin3 protein signal overlap used to calculate the Pearson’s colocalization in Nikon elements. G4C2 RNA:MATR33 Pearson’s coefficient values from 70 RNA foci were binned in values of 10 and colocalization was classified using < − 0.79 inverse colocalization; − 0.8 to 0.19 no colocalization; 0.20 to 0.59 moderate colocalization (yellow, Fig. 1e); 0.8–1 colocalized (green, Fig. 1e).

### Patient-derived induced pluripotent stem cells (iPSCs)

Clinical information and respective sources of all control (healthy) and C9-ALS iPSC lines used in this study are listed in Table 2.

**Table 2:** Summary of iPSC cell lines used in this study

Cell line	Condition	Sex	Age	Reprogramming method	Source/References
18a (Ctrl #1)	Non-neurological control	Female	51	Retrovirus	Boulting et al. [63]; Nat Biotechnol
11a (Ctrl #2)	Non-neurological control	Male	36	Retrovirus	Boulting et al. [63]; Nat Biotechnol
15-12 (Ctrl #3)	Non-neurological control	Male	36	Sendai	RUCDR Infinite Biologics
CS0002 (Ctrl#4)	Non-neurological control	Male	51	Episomal	Cedars Sinai/Answer ALS
C9#3.1 (C9-ALS #1)	C9-ALS	Female	64	Sendai	RUCDR Infinite Biologics
C910689 (C9-ALS #2)	C9-ALS	Female	51	Sendai and episomal	Coriell Institute for Medical Research
C9#2 (C9-ALS #3)	C9-ALS	Female	61	Sendai	RUCDR Infinite Biologics

### iPSC culture and motor neuron differentiation

iPSC differentiation into motor neurons was performed as previously described (55,56). Briefly, iPSCs were dissociated with Accutase and plated at a density of 100,000 cells/cm^2^ in mTESR1 media substituted with a 10 µM ROCK inhibitor (DNSK International, Y-27632). The next day, media was replaced with N2B27 medium (50% DMEM:F12, 50% Neurobasal, supplemented with NEAA, Glutamax, N2 and B27; Gibco) supplemented with SB431542 (10 µM, DNSK International), LDN-193189 (100 nM, DNSK International), Retinoic Acid (1 µM RA, Sigma) and Smoothened-Agonist (1 µM SAG, DNSK International) and fed daily with the same media for 6 days. On day 7, media was switched to N2B27 supplemented with 1 µM RA, 1 µM SAG, 5 µM DAPT (DNSK International) and 4 µM SU5402 (DNSK International) and fed daily with the same media for 7 days. Cell were then dissociated using TrypLE Express (Gibco) supplemented with DNase I (Worthington), plated onto pre-coated Matrigel-coated surfaces (BD Biosciences) and cultured in Neurobasal medium supplemented with NEAA, Glutamax, N2, B27, Ascorbic acid (0.2 µg/mL) and BDNF, CNTF and GDNF (10 ng/mL, R&D systems). For imaging analysis, motor neurons (MNs) were seeded on pre-plated mouse glia cells.

#### siRNA treatments

Two serial siRNA treatments were applied beginning 65–70 days after initiating MN differentiation. Each treatment was applied for 24 h. The second treatment began 4 days after application of the first siRNA treatment and FISH was performed on freshly fixed cells 4 days after the application of the second siRNA treatment.

### Fluorescent in situ hybridization (FISH)

FISH analysis was performed as previously described [42]. Briefly, cells were cultured on 12 mm coverslips in 24 well plates, fixed in 4% PFA, and permeabilized in 0.3% Triton for 15 min. Before hybridization, cells equilibrated in 2× SSC + 50% formamide for 10 min at 60C and probe mixture was preheated for 10 min at 95C. For hybridization, 200 mL of hybridization buffer and 27 mL of probe mixture were added to each coverslip and cells were incubated for 1 h at 60C. Following hybridization, cells were incubated with 2× SSC + 50% formamide for 20 min at 65C, then again with fresh 2× SSC/50% formamide for 15 min at 65C, and finally with 1× SSC + 40% formamide for 10 min at 60C. Cells then underwent a series of washes at RT: 3× quick washes with 1× SSC, 2 × 5 min washes with 1× SSC, a 5 min wash with Tris-buffered saline, and a 5 min wash with Tris–glycine buffer. Cells were post-fixed in 3% PFA and then incubated with blocking buffer for 1 h at RT. Cells incubated with primary antibodies diluted in immunofluorescence buffer overnight at 4C and secondary antibodies diluted in immunofluorescence buffer for 20 min at RT. After secondary antibody incubation, cells were sequentially washed with immunofluorescence buffer, Tris-buffered saline, Tris–glycine buffer, 2 mM MgCl2 in PBS, and PBS. Coverslips were mounted with ProLong Gold Antifade mounting media with DAPI (Invitrogen) and blinded. Hybridization buffer consisted of 40% formamide, BSA (2 mg/mL, Roche), 1 mM ribonucleoside vanadyl complex (Sigma-Aldrich), 10 mM NaPO4, and 1× SSC. Probe mixtures for detection of C9 RNA foci, consisted of 1 mL salmon sperm (10 mg/mL, Thermo Fisher Scientific), 0.5 mL *E. coli* tRNA (20 mg mL, Thermo Fisher Scientific), 25 mL 80% formamide, and 0.4 mL 25 mM 50 digoxigenin (DIG)-labeled CCCCGGCCCCGGCCCC locked nucleic acid probe (Exiqon, batch 620574). Probe mixture for detection of poly (A) RNA, consisted of 1 mL salmon sperm (10 mg/mL, Thermo Fisher Scientific), 0.5 mL *E. coli* tRNA (20 mg mL, Thermo Fisher Scientific), 25 mL 80% formamide, and 0.8 mL 25 mM 30 digoxigenin (DIG)-labeled polyT(25) locked nucleic acid probe (Exiqon, lot 237566815). Tris-buffered saline comprised of 50 mM Tris and 15 mM NaCl solution, pH 7.4. Tris–glycine buffer comprised of 0.75% glycine and 200 mM Tris solution, pH 7.4. Blocking buffer consisted of 1% normal donkey serum (Jackson Immunoresearch) and 5% heat-shocked BSA in Tris-buffered saline. Immunofluorescence buffer consisted of 2% heat-shocked BSA in Tris-buffered saline. DIG-labeled probe was detected with a fluorescein-conjugated sheep anti-DIG antibody (1:250, Roche). All buffers were made with RNase-free water or PBS.

### Immunocytochemistry

Motor neurons, matured for 50 days, and transiently transfected HEK293T cells at 72-h post transfection, were used for immunocytochemistry. Cells were washed and fixed with 4% paraformaldehyde and blocked for 1 h in Phosphate-buffered saline (PBS) containing 0.2% Triton X-100 and 10% normal donkey serum for iPSC-MNs and 5% normal goat serum for HEK293Ts respectively (Jackson ImmunoResearch). Samples were then incubated overnight at 4 °C with primary antibodies. The next day, cells were washed with PBS with 0.1% Triton X-100. Samples were then incubated with the appropriate secondary antibodies (diluted in blocking solution) conjugated to Alexa488, Alexa555 or Alexa647 fluorophores (1:500 to 1:1000 Molecular Probes) for 1 h at RT. Cell nuclei were labeled by DNA staining using DAPI or Hoechst (Life Technologies).

*Primary antibodies* Chicken anti-MAP2 (1:5000, Abcam ab5392); Goat anti-ChAT (1:500); Rabbit anti-MATR3 (1:200; Abcam 151714); Mouse Anti-digoxin (1:200; Jackson Immunoresearch 200-002-156); Rabbit anti-FLAG (1:500, Sigma F7425); Rabbit anti-Histone H3 (1:200; Abcam ab1791)

### Quantitative image acquisition and analysis

Images used for quantification were acquired at matched exposure times or laser settings and processed using identical settings. Quantifications were normalized within each respective experiment with n = 3 independent experiments unless otherwise specified in figure legends. Image acquisition was performed on a Leica DMI4000B laser scanning confocal microscope (Leica, Buffalo Grove, IL) or a Nikon W1 dual camera spinning disk confocal microscope (Northwestern University Center for Advanced Microscopy). Unless otherwise specified, image acquisition was performed through the z-dimension at 0.3–0.5 mm intervals and individual planes were projected into maximum intensity images.

#### Quantification of nuclear MATR3 and nuclear Histone H3 immunoreactivity

The nuclear region of interest was defined by DAPI and the mean signal intensity of MATR3 as the mean pixel intensity per mm^2^ as calculated by the NIS-Elements Advanced Research v5 software (Nikon software, Northwestern University Center for Advanced Microscopy). Fold change was determined by averaging the values from all control neurons and normalizing to this average.

#### Quantification of G4C2 RNA foci

FISH images were acquired in a blinded manner using a Plan Apo l 60× 1.45 NA oil immersion objective through the z dimension at 0.3 mm intervals and set to equal contrast. Images were cropped to single neuron dimensions and analyzed by individual plane for nuclear G4C2 foci in a blinded manner by the primary operator in one sitting. We defined “G4C2 foci” as signals that met the following criteria: (a) diameter > 0.8 µm (b) signal intensity > 1.5 fold higher than diffuse nuclear signal (c) punctate morphology and (d) nuclear localization.

#### MATR3 and G4C2 colocalization analysis

The assessment of the % of G4C2 RNA foci that colocalized with MATR3 was performed by identifying nuclear G4C2 foci as described above and tested for the presence or absence of total MATR3 signal (punctuate and diffuse). The assessment of the % of MATR3 puncta that colocalized with G4C2 RNA foci was performed by identifying nuclear MATR3 puncta (based on size and contrast relative to overall nuclear MATR3 signal intensity) and tested for the presence or absence of a G4C2 RNA focus.

### Drosophila assays

The detailed list of *Drosophila* lines used in this study and their respective sources are outlined in Table 3. All *Drosophila* stocks were cultured on standard dextrose media on a 12-h light/dark cycle.

**Table 3:** List of *Drosophila* lines used in this study

Line	Source
*Transgenic lines*	
UAS-MATR3	Ramesh et al. [64]; Acta Neuropath. Comm.
UAS-MATR3-ΔRRM1	Ramesh et al. [64]; Acta Neuropath. Comm.
UAS-MATR-ΔRRM2	Ramesh et al. [64]; Acta Neuropath. Comm.
UAS-MATR3-ΔZNF1	Ramesh et al. [64]; Acta Neuropath. Comm.
UAS-MATR3-ΔZNF2	Ramesh et al. [64]; Acta Neuropath. Comm.
UAS-TDP43	Gift from Dr. Paul Taylor
UAS-FUS	Lanson et al. [65]; Human Mol Genet
UAS-EWSR1	Gift from Dr. Nancy Bonini
UAS-G4C2-3R	Xu et al. [36]; PNAS
UAS-G4C2-30R	Xu et al. [36]; PNAS
UAS-G4C2-58R	Freibaum et al. [35]; Nature
UAS-G4C2-36R	Mizielinska et al. [37]; Science
UAS-GR36	Mizielinska et al. [37]; Science
UAS-PR36	Mizielinska et al. [37]; Science
UAS-GA36	Mizielinska et al. [37]; Science
UAS-GP36	Mizielinska et al. [37]; Science
*Driver lines*	
GMR-Gal4	Bloomington Stock Center #1104
OK371-Gal4	Bloomington Stock Center #26160
ElavGS-Gal4	Gift from Dr. Haig Keshishian

### Eye degeneration quantification

Expression of transgene in *Drosophila* eyes was driven using the GMR-gal4 driver at 28 °C. One day old F1 adults (or 30-day old adults for aging experiments) were collected and external images of the eye were using Leica M205C microscope equipped with a Leica DFC450 camera. Percent external eye degeneration was quantified in an unblinded manner using a previously published scoring system [53, 57, 58, 60].

### Adult survival and motor dysfunction

For conditional pan-neuronal expression, G4C2-30R and UAS-MATR3 lines were crossed with inducible driver, ElavGS-Gal4. Day 1 adults from the F1 progeny were collected every 24 h and moved to standard media mixed with 20 mM RU486 to induce transgene expression in adults. The adults were cultured at 28 °C and death was recorded every other day. Motor function was assessed on day-30 of their lifespan. Locomotion was assessed using the RING assay, as previously described [61] with a few modifications. Briefly, flies were transferred, without anesthetization, into plastic vials and placed in the RING apparatus. The vials were tapped down against the bench and the climbing was recorded on video. Quantifications were performed manually by a third party in a blinded manner.

### Plasmids

FLAG-tagged MATR3, ΔRRM1, ΔRRM2, ΔZNF1 and ΔZNF2 in pCMV-Tag2B mammalian expression vector were a gift from Yossi Shiloh (Addgene plasmid #32880, 32881. 32882, 32883, 32884). For generating *Drosophila* lines, FLAG-MATR3 constructs were cloned into pUASTaTTB vector between NotI-XhoI restriction sites. All sequences were verified by Sanger sequencing. The pcDNA3.0-IRES2X-G4C2_60_-Dendra2 construct was a generous gift from Dr. Aaron Haeusler at Thomas Jefferson University. Ambion *Silencer*™ Select siRNA for MATR3 (s18897) and scrambled negative control (4390843) were obtained from Thermofisher.

### Cell culture and transfections

HEK293T cells (ATCC^®^ CRL-3216™) were cultured in Advanced DMEM supplemented with 10% FBS and 1% Glutamax and grown at 37 °C and 5% CO_2_. HEK293T cells were transiently transfected using Lipofectamine 3000 (Invitrogen L3000001) following manufacturer instructions.

### Biotinylated G4C2 RNA pull-down

G4C2_10_ oligomer biotinylated at the 5′ end was synthesized by Integrated DNA Technologies Inc. (IDT) and RNA pull-down was performed as previously described [25]. Nuclear lysates from HEK293T cells were extracted using the NE-PER nuclear cytoplasmic extraction kit (ThermoScientific 78833) following manufacturer instructions. 0.5 mg of lysates made up to 500 µL with in RNA structure buffer (10 mM Tris pH 7, 0.1 M KCl, 10 mM MgCl_2_) were pre-cleared with 50 µL of pre-washed Dynabeads Streptavidin Magnetic Beads (Thermofisher 11205D) at 4 °C overnight on a rotary shaker. 4 µg of biotinylated RNA was heated to 90 °C for 2 min in RNA structure buffer (10 mM Tris pH 7, 0.1 M KCl, 10 mM MgCl2) and incubated at for 20 min at RT. Biotin-G4C2_10_ RNA oligomer was added to the pre-cleared nuclear extract and incubated at 4 °C for 4 h. 50 µL of pre-washed Dynabeads Streptavidin Magnetic Beads was added to each reaction and incubated at 4 °C for 1 h. After incubation with the nuclear extracts, beads were washed 5 times in wash buffer (10 mM Tris–Cl, pH 7.5; 1 mM EDTA, 2 M NaCl). Co-precipitated proteins were eluted from bound biotinylated RNAs in wash buffer and boiled in 1× NuPage LDS-Sample buffer (Invitrogen NP0007) at 95 °C for 5 min. Co-precipitated proteins were detected by Western Blot. All buffers were freshly supplemented with 0.5 mM DTT, 0.1 U/mL RNase inhibitor, 0.1 µg/µL tRNA and 5 mM EDTA.

### Preparation of lysates for SDS-PAGE and dot blot

*Drosophila* tissue was first flash frozen on dry ice and crushed using pestles. For preparation of lysate, crushed tissue or cells were incubated in RIPA buffer: 150 mM NaCl, 1% NP40, 0.1% SDS, 1% sodium deoxycholate, 50 mM NaF, 2 mM EDTA, 1 mM DTT, 2.5 mM Na orthovanadate, 1× protease inhibitor cocktail (Roche 11836170001), pH 7.4. The samples were sonicated in an ultrasonic bath and centrifuged down at 12,000×*g* for 10 min. The supernatant was boiled in 1× NuPage LDS-Sample buffer (Invitrogen NP0007) at 95 °C for 5 min.

Primary antibodies: mouse anti-FLAG (1:1000; Sigma F1804); mouse anti-α tubulin (1:8000; Sigma T5168)

### RNA preparation and RT-quantitative PCR (qPCR)

RNA was isolated using RNeasy Mini Kit (Qiagen) according to manufacturer instructions. cDNA synthesis was performed using the iScript Select cDNA Synthesis Kit (BioRad; 170-8897) and was subsequently run using the Bio-Rad iQ™ Supermix (170-8862) on a 96-well plate (Applied Biosystems, #4306737) on Applied Biosystems StepOnePlus Real-Time PCR system. Primer pairs and probes were designed for each target of interest and housekeeping control α-tubulin (for *Drosophila*) and GAPDH (for human) using Integrated DNA Technologies PrimeTime qPCR Assay (www.idtdna.com). The comparative Ct method was used for analyzing the fold change differences [62]. The list of primer–probe sequences are listed in Table 4.

**Table 4:** List of primer and probe sequences (5′–3′) used for real-time quantitative PCR (RT-qPCR)

*α*-*tubulin* (*Drosophila*)	
Primer 1	ACCAGCCTGACCAACATG
Primer 2	CCTCGAAATCGTAGCTCTACAC
Probe	/56FAM/TCACACGCG/ZEN/ACAAGGAAAATTCACAGA/3IABkFQ/
*MATR3* (human)	
Primer 1	CTTCTTCTGTCTGCGTTCTTCT
Primer 2	TACTGTAAGCTGTGTTCACTCTT
Probe	/56FAM/ACTCATTGC/ZEN/AGCAGCCTTCCTCA/3IABkFQ/
*GAPDH* (human)	
Primer 1	GTGGAGTCATACTGGAACATGTAG
Primer 2	AATGGTGAAGGTCGGTGTG
Probe	/56-FAM/TGCAAATGG/ZEN/CAGCCCTGGTG/3IABkFQ/
*Dendra2*	
Primer 1	ACTTCAAGCAGAGCTTCCC
Primer 2	ACGTTCTGGAAGAAGCAGTC
Probe	/56-FAM/CAGATGCCC/ZEN/TTGTCCTCGAAGGTC/3IABkFQ/
*GFP*	
Primer 1	GAACCGCATCGAGCTGAA
Primer 2	TGCTTGTCGGCCATGATATAG
Probe	/56-FAM/ATCGACTTC/ZEN/AAGGAGGACGGCAAC/3IABkFQ/
*HIST1H3D*	
Primer 1	GGACTTGCGAGCAGTCTG
Primer 2	CATCCAGGCTGTACTGCT
Probe	/56-FAM/CCTTGGCCT/ZEN/TGTGGTGACTCTCA/3IABkFQ/

### Statistical analysis

All statistical analyses were performed on GraphPad Prism6.

## Supplementary information

Additional file 1: Figure S1. Control iPSC-derived motor neurons and post-mortem brain tissues are negative for G4C2 foci. (**A**) Representative confocal images of control iPSC-MN indicated by MAP2 (gray). White dotted box represents area of high magnification in the images on the right. FISH-IF staining of G4C2 RNA foci (green) and MATR3 (red) showed no signal for G4C2 foci in control iPSC neurons, and thus no colocalization between MATR3 puncta (yellow arrow) and G4C2. (**B**) FISH-IF of control post-mortem brains are negative for G4C2 foci. White-dotted circle demarcates the nucleus. Asterisks denote autofluorescence due to lipofuscin

Additional file 2: Figure S2. G4C2 colocalization with punctate and diffuse MATR3 in C9-ALS iPSC MNs. (**A**) Representative confocal images of C9-ALS patient-derived iPSCs that were differentiated to neurons (represented by MAP2, gray) showing colocalization between G4C2 RNA foci (green) with MATR3 (red) in diffuse form (yellow arrows). Dotted-white boxes show G4C2 foci represented in the high-magnification panels on the right (**B**) The percentage distribution of MATR3 puncta that co-localize with G4C2 foci (blue) and are independent of G4C2 foci (red) in two independent C9-ALS iPSC MNs (C9-ALS #1 and C9-ALS #2) and the combined average

Additional file 3: Figure S3. Levels of nuclear Histone H3 not altered in C9-ALS patient neurons. (**A**) Representative confocal images of C9-ALS iPSC differentiated to neurons (represented by MAP2, red) stained for Histone H3 nuclear protein (green/gray) (**B**) Quantification of endogenous Histone H3 immunofluorescence levels in MAP2^+^ neurons revealed no significant differences in the levels of nuclear Histone H3 in C9-ALS iPSC-MNs compared to that in control iPSC-MNs (Unpaired *t* test) n = 3 (**C**) Quantification of *HIST1H3D* mRNA fold change in control and C9-ALS iPSC MNs showed no significant differences in the mRNA levels in C9-ALS iPSC-MNs compared to that in control (Unpaired t-test) n = 10-12. Error bars indicate S.E.M

Additional file 4: Figure S4. MATR3-mediated suppression G4C2-30R toxicity retained upon aging. (**A**) Representative images of *Drosophila* eyes from flies expressing G4C2-30R along with UAS-EGFP (control for GAL4 dilution), UAS-MATR3, or (**B**) UAS-MATR3-ΔRRM2. Flies expressing G4C2-30R develop exacerbated eye degeneration upon aging, indicated by increase in de-pigmentation, ommatidial fusion and development of new necrotic patches. Ectopic expression of MATR3 continued to strongly suppress eye degeneration upon aging. Ectopic expression of MATR3-ΔRRM2 also suppressed eye degeneration in G4C2-30R flies at 30-days, however to a much lesser extent compared to full-length MATR3. (**C**) Quantification of external eye degeneration showed statistically significant suppression of G4C2-30R- mediated eye degeneration upon MATR3 expression, and to a milder extent, upon MATR3-ΔRRM2 expression (Kruskal–Wallis test) n ≥ 50 flies per genotype. Error bars indicate S.E.M. ****p value < 0.0001

Additional file 5: Figure S5. Ectopic expression of TDP43, FUS and EWSR1 do not suppress G4C2-30R toxicity in *Drosophila*. (**A**) Representative images and (**B**) quantification of external eye degeneration of *Drosophila* eyes from flies expressing RNA-binding proteins TDP43, FUS and EWSR1 on its own (top) or co-expressed with G4C2-30R (bottom). Expression of either TDP43, FUS or EWSR1 on its own resulted in external eye degeneration (**A**, **top**), that was further exacerbated when co-expressed with G4C2-30R (**A**, **bottom**) suggesting compounded toxicities. n ≥ 50 flies per genotype. Error bars indicate S.E.M. ***p value < 0.001; ****p value < 0.0001

Additional file 6: Figure S6. Deletion in functional domains of MATR3 do not cause external eye degeneration. (**A**) Representative western blot of full-length MATR3 and deletion variants: ΔRRM1, ΔRRM2, ΔZF1 and ΔZF2. α-tubulin was used as loading control. Deletion variants are lower in size compared to full-length MATR3. (**B**) Representative images *Drosophila* eyes from flies expressing MATR3 deletion variants that showed normal (non-degenerative) eye phenotype

Additional file 7: Figure S7. Validation of siRNA mediated knockdown of *MATR3*. (**A**) Representative western blot of endogenous MATR3 in HEK293T cells that have been treated with either scrambled control siRNA or MATR3 siRNA. (**B**) Quantification of 3 independent blots showed statistically significant reduction of MATR3 protein levels in cells treated with *MATR3* siRNA compared to scrambled control. (Mann–Whitney U-test). (**C**) Representative confocal images of C9-ALS patient derived iPSC-MNs (indicated by MAP2, gray) transfected with either scrambled siRNA or *MATR3* siRNA and stained for MATR3 (red)

## Abbreviations

ALS: amyotrophic lateral sclerosis
C9orf72: chromosome 9 open reading frame 72
HRE: hexanucleotide repeat expansion
DPR: dipeptide protein repeat
RBP: RNA-binding protein
RAN: repeat-associated non-ATG (translation)
RRM: RNA-recognition motif
ZF: zinc finger
FXTAS: fragile X tremor ataxia syndrome
SCA31: Spinocerebellar ataxia type-31
iPSC: induced pluripotent stem cell
MN: motor neuron
NLS: nuclear localization signal
FISH: Fluorescent in situ hybridization
IF: immunofluorescence
IHC: immunohistochemistry
ICC: immunocytochemistry
RT qPCR: real-time quantitative polymerase chain reaction
