# Supplementary figures and images for: RNA dependent suppression of C9orf72 ALS/FTD associated neurodegeneration by Matrin-3
Source: Acta Neuropathol Commun. 2020 Oct 31;8:177. doi: 10.1186/s40478-020-01060-y (PMC7603783; doi:10.1186/s40478-020-01060-y)

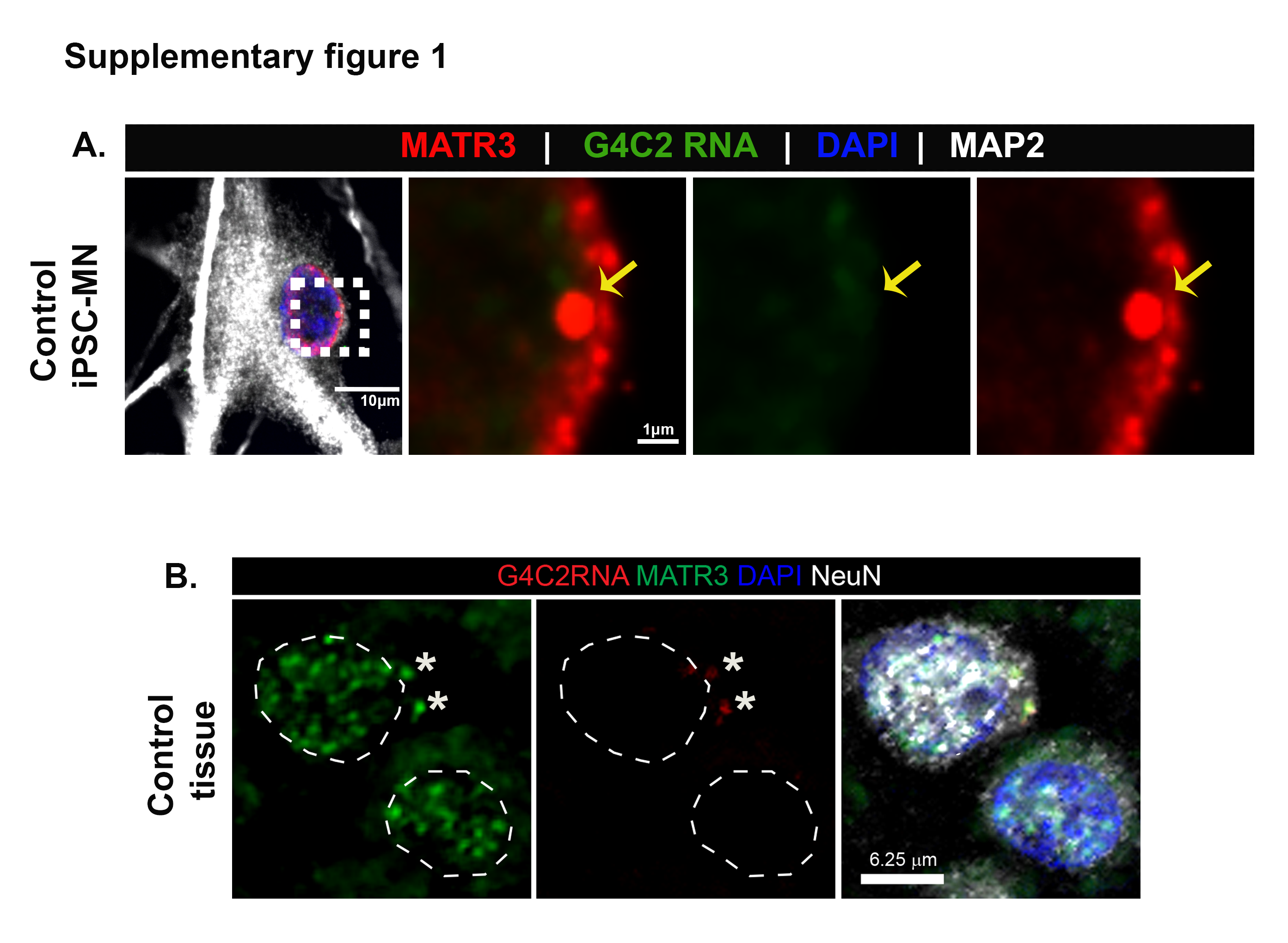

Supplement: Supplementary file 1 — Control iPSC-derived motor neurons and post-mortem brain tissues are negative for G4C2 foci. (A) Representative confocal images of control iPSC-MN indicated by MAP2 (gray). White dotted box represents area of high magnification in the images on the right. FISH-IF staining of G4C2 RNA foci (green) and MATR3 (red) showed no signal for G4C2 foci in control iPSC neurons, and thus no colocalization between MATR3 puncta (yellow arrow) and G4C2. (B) FISH-IF of control post-mortem brains are negative for G4C2 foci. White-dotted circle demarcates the nucleus. Asterisks denote autofluorescence due to lipofuscin [file 40478_2020_1060_MOESM1_ESM.tif]

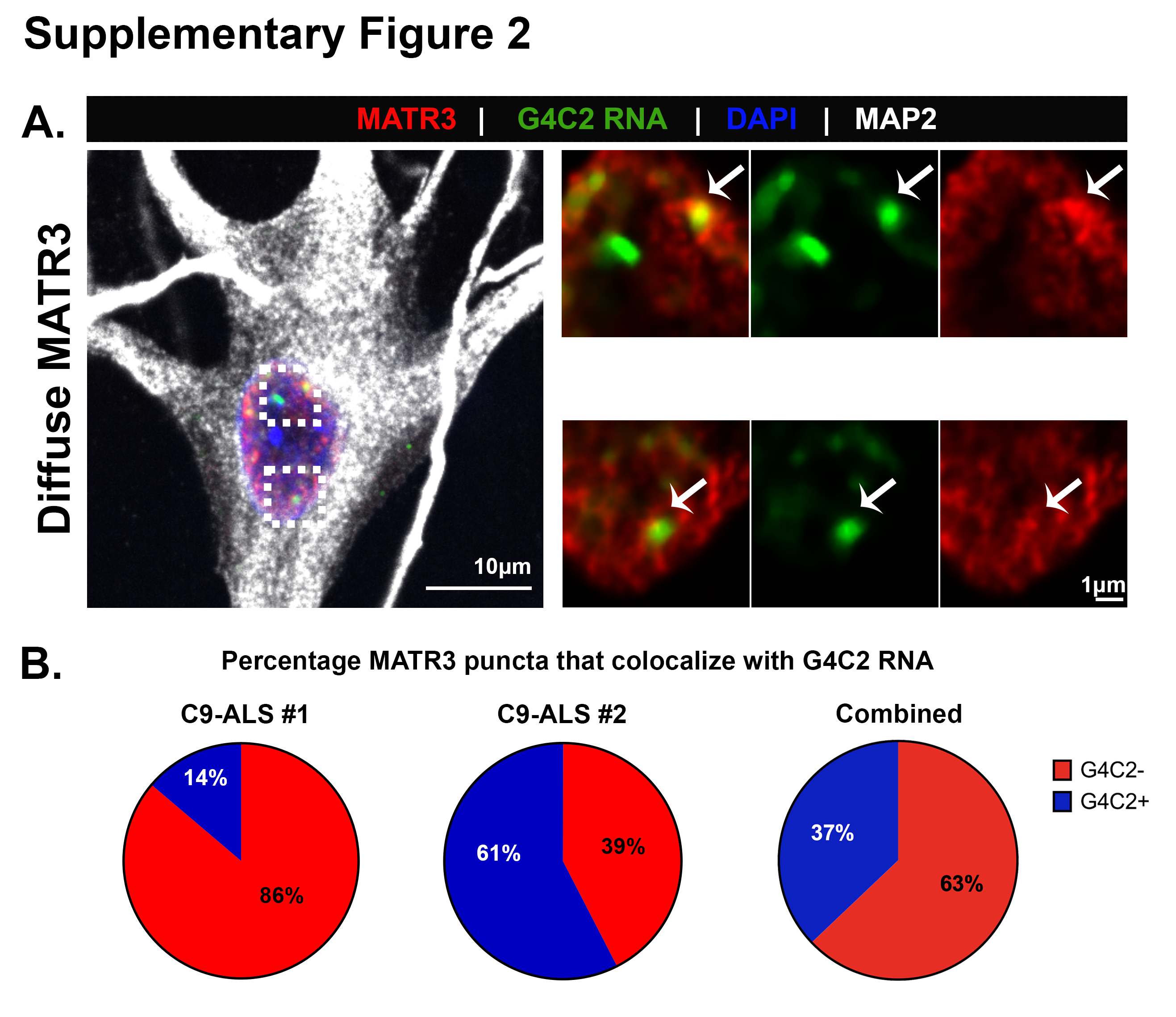

Supplement: Supplementary file 2 — G4C2 colocalization with punctate and diffuse MATR3 in C9-ALS iPSC MNs. (A) Representative confocal images of C9-ALS patient-derived iPSCs that were differentiated to neurons (represented by MAP2, gray) showing colocalization between G4C2 RNA foci (green) with MATR3 (red) in diffuse form (yellow arrows). Dotted-white boxes show G4C2 foci represented in the high-magnification panels on the right (B) The percentage distribution of MATR3 puncta that co-localize with G4C2 foci (blue) and are independent of G4C2 foci (red) in two independent C9-ALS iPSC MNs (C9-ALS #1 and C9-ALS #2) and the combined average [file 40478_2020_1060_MOESM2_ESM.tif]

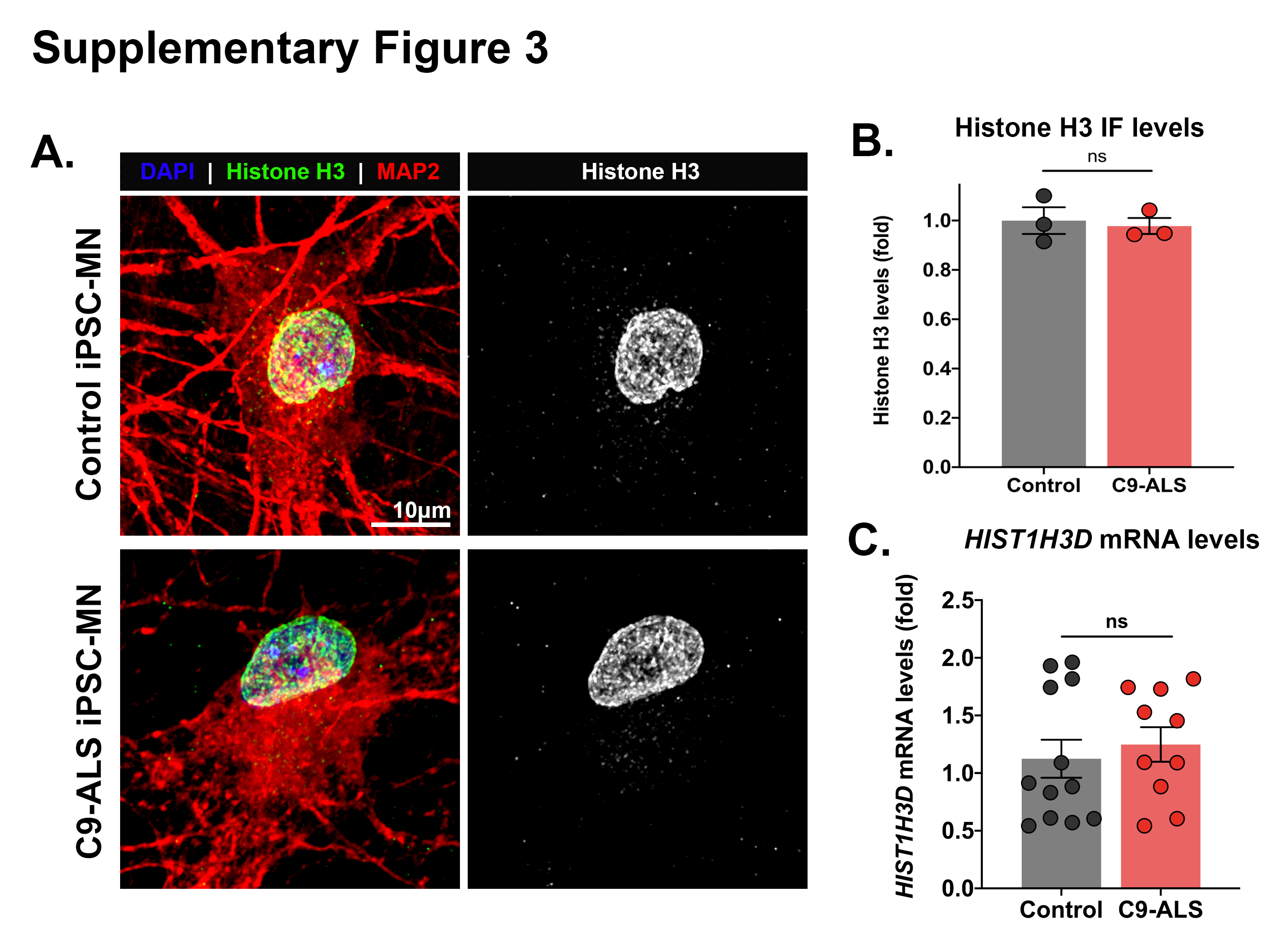

Supplement: Supplementary file 3 — Levels of nuclear Histone H3 not altered in C9-ALS patient neurons. (A) Representative confocal images of C9-ALS iPSC differentiated to neurons (represented by MAP2, red) stained for Histone H3 nuclear protein (green/gray) (B) Quantification of endogenous Histone H3 immunofluorescence levels in MAP2+ neurons revealed no significant differences in the levels of nuclear Histone H3 in C9-ALS iPSC-MNs compared to that in control iPSC-MNs (Unpaired t test) n = 3 (C) Quantification of HIST1H3D mRNA fold change in control and C9-ALS iPSC MNs showed no significant differences in the mRNA levels in C9-ALS iPSC-MNs compared to that in control (Unpaired t-test) n = 10-12. Error bars indicate S.E.M [file 40478_2020_1060_MOESM3_ESM.tif]

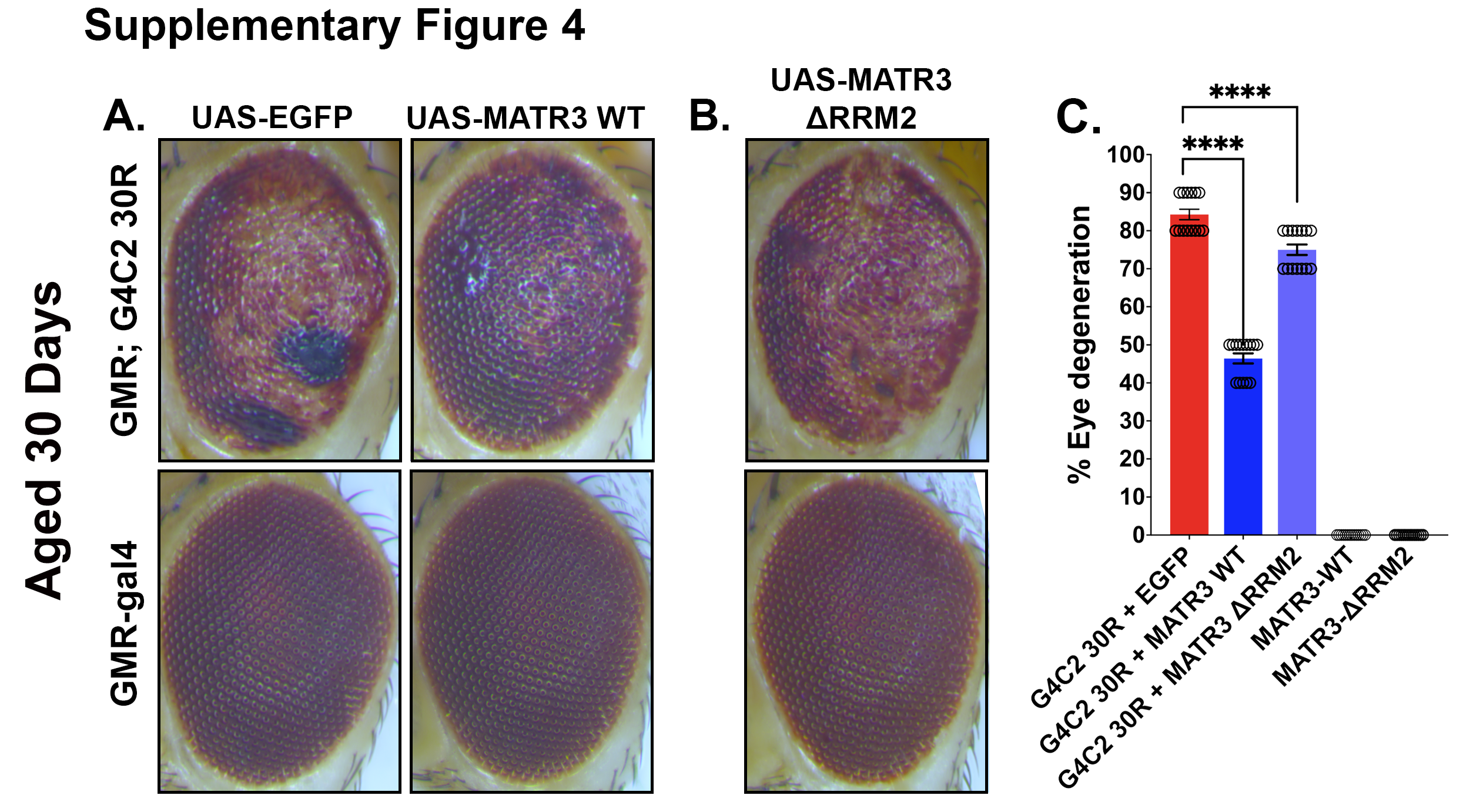

Supplement: Supplementary file 4 — MATR3-mediated suppression G4C2-30R toxicity retained upon aging. (A) Representative images of Drosophila eyes from flies expressing G4C2-30R along with UAS-EGFP (control for GAL4 dilution), UAS-MATR3, or (B) UAS-MATR3-ΔRRM2. Flies expressing G4C2-30R develop exacerbated eye degeneration upon aging, indicated by increase in de-pigmentation, ommatidial fusion and development of new necrotic patches. Ectopic expression of MATR3 continued to strongly suppress eye degeneration upon aging. Ectopic expression of MATR3-ΔRRM2 also suppressed eye degeneration in G4C2-30R flies at 30-days, however to a much lesser extent compared to full-length MATR3. (C) Quantification of external eye degeneration showed statistically significant suppression of G4C2-30R- mediated eye degeneration upon MATR3 expression, and to a milder extent, upon MATR3-ΔRRM2 expression (Kruskal–Wallis test) n ≥ 50 flies per genotype. Error bars indicate S.E.M. ****p value < 0.0001 [file 40478_2020_1060_MOESM4_ESM.tif]

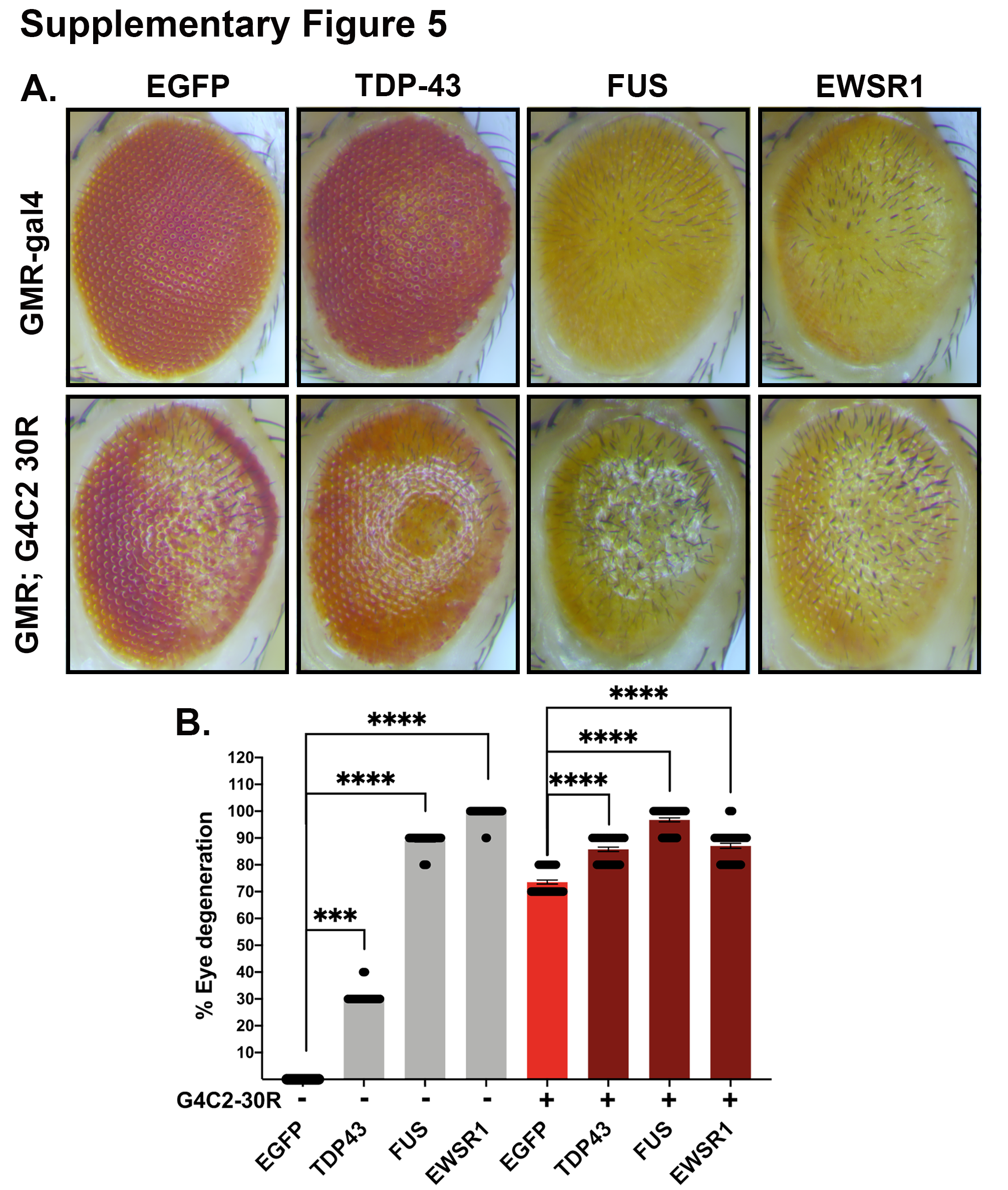

Supplement: Supplementary file 5 — Ectopic expression of TDP43, FUS and EWSR1 do not suppress G4C2-30R toxicity in Drosophila. (A) Representative images and (B) quantification of external eye degeneration of Drosophila eyes from flies expressing RNA-binding proteins TDP43, FUS and EWSR1 on its own (top) or co-expressed with G4C2-30R (bottom). Expression of either TDP43, FUS or EWSR1 on its own resulted in external eye degeneration (A, top), that was further exacerbated when co-expressed with G4C2-30R (A, bottom) suggesting compounded toxicities. n ≥ 50 flies per genotype. Error bars indicate S.E.M. ***p value < 0.001; ****p value < 0.0001 [file 40478_2020_1060_MOESM5_ESM.tif]

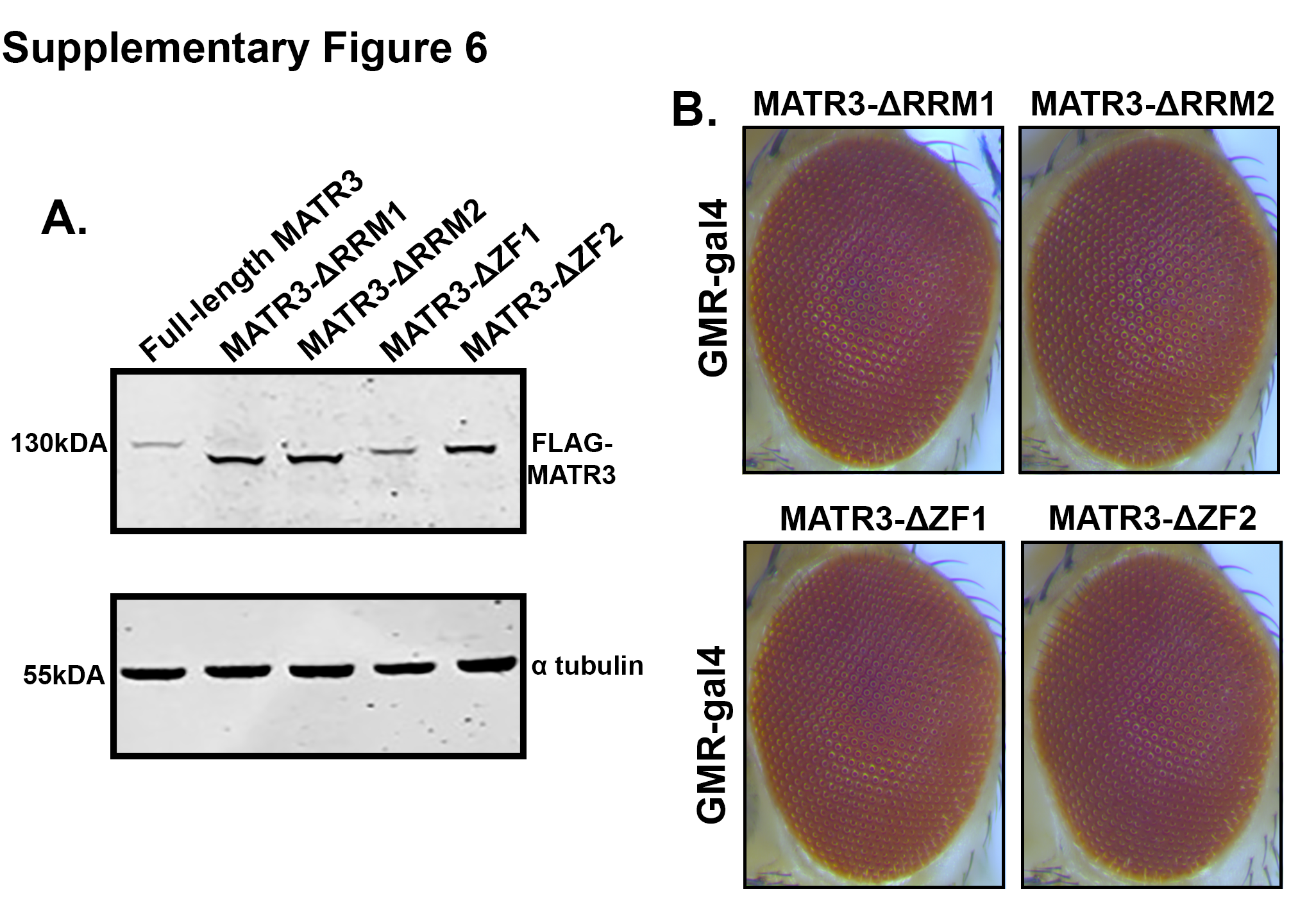

Supplement: Supplementary file 6 — Deletion in functional domains of MATR3 do not cause external eye degeneration. (A) Representative western blot of full-length MATR3 and deletion variants: ΔRRM1, ΔRRM2, ΔZF1 and ΔZF2. α-tubulin was used as loading control. Deletion variants are lower in size compared to full-length MATR3. (B) Representative images Drosophila eyes from flies expressing MATR3 deletion variants that showed normal (non-degenerative) eye phenotype [file 40478_2020_1060_MOESM6_ESM.tif]

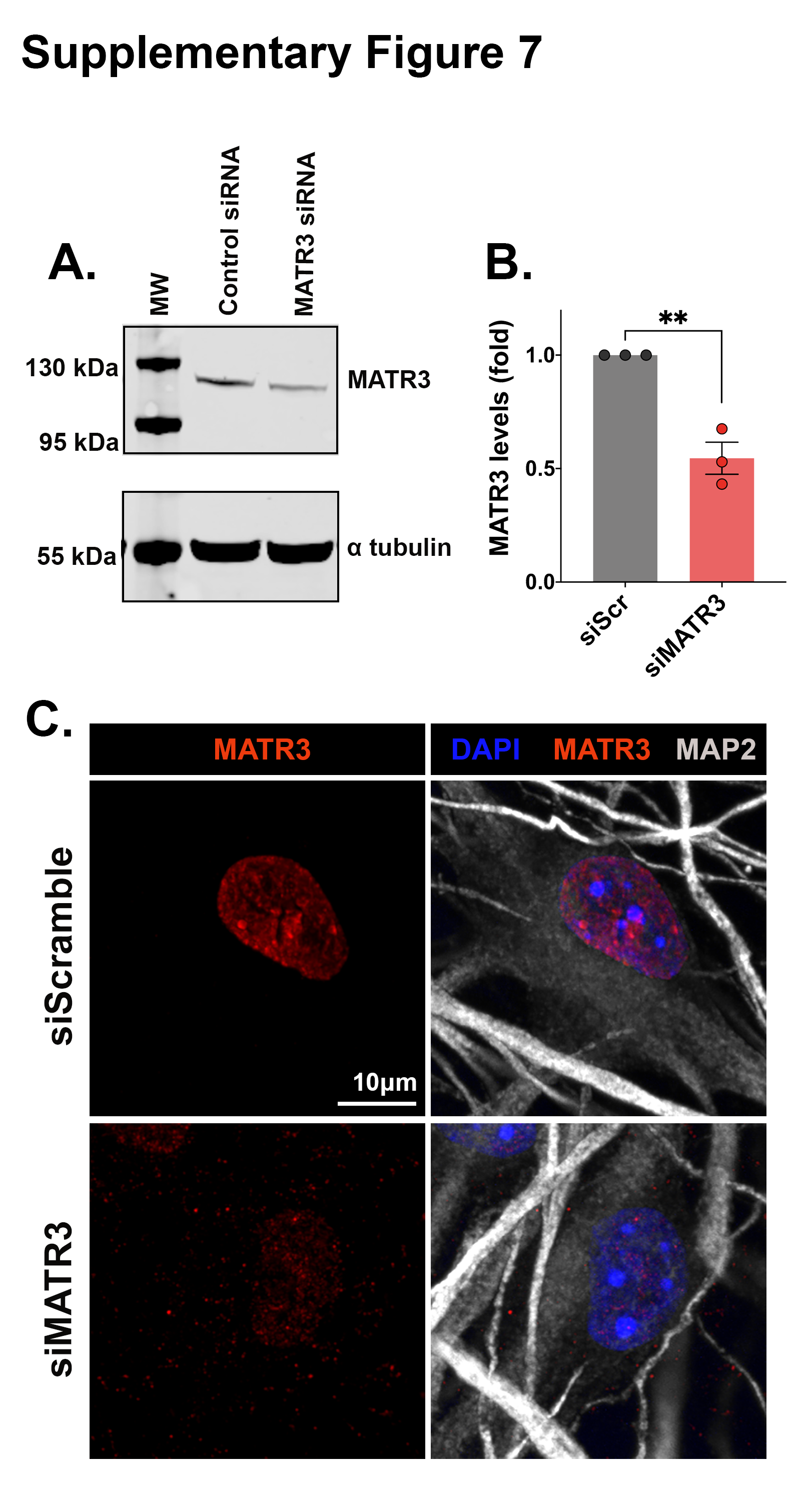

Supplement: Supplementary file 7 — Validation of siRNA mediated knockdown of MATR3. (A) Representative western blot of endogenous MATR3 in HEK293T cells that have been treated with either scrambled control siRNA or MATR3 siRNA. (B) Quantification of 3 independent blots showed statistically significant reduction of MATR3 protein levels in cells treated with MATR3 siRNA compared to scrambled control. (Mann–Whitney U-test). (C) Representative confocal images of C9-ALS patient derived iPSC-MNs (indicated by MAP2, gray) transfected with either scrambled siRNA or MATR3 siRNA and stained for MATR3 (red) [file 40478_2020_1060_MOESM7_ESM.tif]
